# Downregulation of rRNA synthesis by BCL-2 induces chemoresistance in diffuse large B cell lymphoma

**DOI:** 10.1016/j.isci.2025.112333

**Published:** 2025-04-02

**Authors:** Alessandra Rossi, Saveria Mazzara, Dorotea Salemi, Simone Zanetti, Maria Rosaria Sapienza, Stefania Orecchioni, Giovanna Talarico, Paolo Falvo, Alessandro Davini, Claudio Ceccarelli, Giovanna Motta, Federica Melle, Valentina Tabanelli, Claudio Agostinelli, Davide Trerè, Marianna Penzo, Chiara Corsini, Elena Baiardi, Angelica Calleri, Umberto Vitolo, Francesco Bertolini, Pier Luigi Zinzani, Roberto Chiarle, Corrado Tarella, Stefano Pileri, Enrico Derenzini

**Affiliations:** 1 Oncohematology Division, IEO European Institute of Oncology IRCCS, Milan, Italy; 2 Division of Diagnostic Haematopathology, IEO European Institute of Oncology IRCCS, Milan, Italy; 3 Department of Computing Sciences and Bocconi Institute for Data Science and Analytics (BIDSA), Bocconi University, Milan, Italy; 4 AI and Systems Biology, IFOM, ETS, Milan, Italy; 5 Laboratory of Hematology-Oncology, IEO European Institute of Oncology IRCCS, Milan, Italy; 6 Department of Medical and Surgical Sciences (DIMEC), Alma Mater Studiorum University of Bologna, Bologna, Italy; 7 Haematopathology Unit, IRCCS Azienda Ospedaliero-Universitaria of Bologna, Bologna, Italy; 8 Department Program in Laboratory Medicine, IRCCS Azienda Ospedaliero-Universitaria of Bologna, Bologna, Italy; 9 Center for Applied Biomedical Research (CRBA), Alma Mater Studiorum University of Bologna, Bologna, Italy; 10 Multidisciplinary Oncology Outpatient Clinic, Candiolo Cancer Institute, FPO-IRCCS, Candiolo, Italy; 11 Seràgnoli Hematology Institute, IRCCS AOU (Azienda Ospedaliero-Universitaria) of Bologna, Bologna, Italy; 12 Boston Children’s Hospital, Department of Pathology, Harvard Medical School, Boston, MA, USA; 13 Department of Molecular Biotechnology and Health Sciences, University of Torino, Turin, Italy; 14 Department of Health Sciences, University of Milan, Milan, Italy

**Keywords:** Cell biology, Cancer

## Abstract

Overexpression of the antiapoptotic oncogene *BCL-2* predicts poor prognosis in diffuse large B cell lymphoma (DLBCL) treated with anthracycline-based chemoimmunotherapy. Anthracyclines exert antitumor effects by multiple mechanisms including inhibition of ribosome biogenesis (RiBi) through rRNA synthesis blockade. RiBi inhibitors induce p53 stabilization through the ribosomal proteins-MDM2-p53 pathway, with stabilized p53 levels depending on baseline rRNA synthesis rate. We found that the BH3-mimetic venetoclax could not fully reverse BCL-2-mediated resistance to RiBi inhibitors in DLBCL cells. BCL-2 overexpression was associated with decreased baseline rRNA synthesis rate, attenuating p53 stabilization by RiBi inhibitors. Drugs stabilizing p53 irrespective of RiBi inhibition reversed BCL-2-induced resistance *in vitro* and *in vivo*, restoring p53 activation and apoptosis. A small nucleolar size, indicative of low baseline rRNA synthesis, correlated with high BCL-2 levels and poor outcomes in DLBCL patients. These findings uncover alternative BCL-2-dependent chemoresistance mechanisms, providing a rationale for specific combination strategies in BCL-2 positive lymphomas.

## Highlights

• Inhibition of rRNA synthesis is a relevant mechanism of anthracycline cytotoxicity
• BCL-2 overexpression is associated with a decreased rRNA synthesis rate in DLBCL
• Impaired baseline rRNA synthesis attenuates p53 activation by RiBi inhibitors
• Combinations restoring p53 activity overcome BCL-2 mediated RiBi inhibitor resistance

## Graphical abstract

**Figure:**
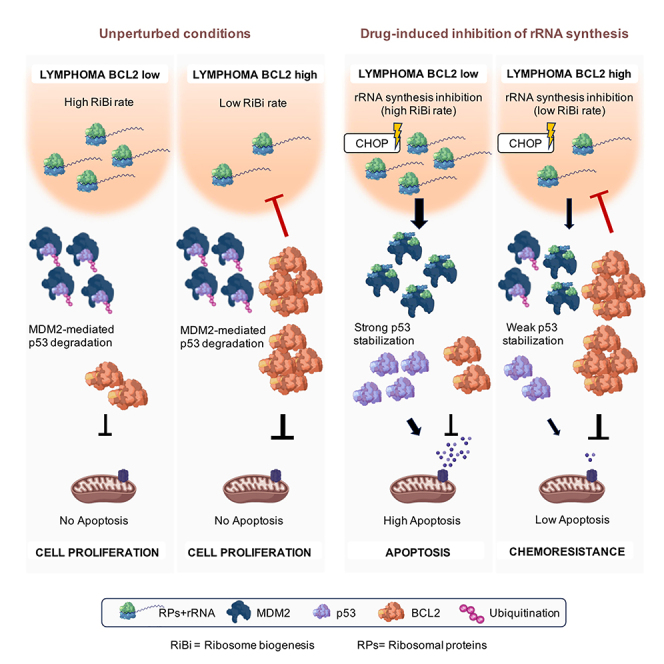

## Introduction

The CHOP (cyclophosphamide, doxorubicin, vincristine, and prednisone) chemotherapy regimen has been the cornerstone of diffuse large B cell lymphoma (DLBCL) first-line induction chemotherapy for three decades. However, even with the addition of the anti CD20 antibody rituximab, a significant fraction of DLBCL patients (30%–40%) are refractory or relapse after first-line treatment.^1^

Anthracyclines and cyclophosphamide exert their anticancer activity by multiple mechanisms; however, a major contribution to their cytostatic and cytotoxic effects involves the inhibition of rRNA transcription and induction of murine double minute 2 (MDM2)-dependent p53 stabilization and activation.^2^^,^^3^ In fact, following inhibition of rRNA synthesis, ribosomal proteins (RPs), which are no longer used for building new ribosomes, are left free to bind and inactivate MDM2, thus blocking p53 protease digestion,^4^^,^^5^ leading to p53 stabilization. Therefore, ribosome biogenesis (RiBi) inhibition with activation of the RP/MDM2/p53 pathway is an important mechanism that contributes to the anti-lymphoma activity of currently used chemotherapeutic drugs.^6^^,^^7^^,^^8^ Notably, the induction of apoptotic cell death depends on the amount of p53 stabilization—lower levels of p53 result in cell-cycle arrest, whereas higher levels result in apoptotic cell death.^9^ Despite these notions, and although a direct relationship between the RiBi rate and efficacy of RiBi inhibitors has been demonstrated in preclinical models,^10^^,^^11^ the correlation between the baseline RiBi rate and DLBCL outcome following chemoimmunotherapy treatment has not been investigated.

Although the occurrence of TP53 mutations could be a potential mechanism of resistance to chemotherapy-induced RP/MDM2/p53 pathway activation, the majority of DLBCL patients display wild-type (WT) TP53,^12^ thus indicating that alternative mechanisms of resistance could explain the failure of anthracycline-containing regimens in a significant fraction of cases.^13^^,^^14^^,^^15^

Several studies have demonstrated that overexpression or rearrangements of *MYC* and *BCL-2* oncogenes are adverse prognostic predictors, identifying a patient population with intrinsic resistance to standard chemoimmunotherapy.^16^

In recent years, *BCL-2* has emerged as the main driver of poor prognosis in DLBCL. While *BCL-2* expression has been repeatedly reported as a poor prognostic predictor, the additive value of *MYC* overexpression seems to be context-dependent. Consistent with this, *BCL-2* overexpression has been described as an independent adverse prognostic predictor irrespective of cell of origin (COO) subtyping and *MYC* expression levels.^17^^,^^18^^,^^19^^,^^20^^,^^21^^,^^22^

Due to its role in B cell lymphomagenesis and chemoresistance, BCL-2 has been considered an attractive therapeutic target in B cell malignancies.^23^^,^^24^^,^^25^

Despite the established role of BCL-2 as a predictor of chemoresistance in DLBCL, specific inhibition of its anti-apoptotic activity by the BH3-mimetic venetoclax showed low efficacy in the relapsed/refractory (r/r) setting.^26^ Moreover, venetoclax has been investigated in combination with R-CHOP chemoimmunotherapy as a first-line treatment for DLBCL, with suboptimal results.^27^^,^^28^

In this study, we first observed that in DLBCL cell lines, BCL-2 overexpression promoted resistance to drugs inhibiting rRNA synthesis (doxorubicin, actinomycin D [Act D], and CX-5461). However, the addition of the BH3 mimetic venetoclax could not fully reverse BCL-2 mediated resistance to RiBi inhibitors. We demonstrate that enforced BCL-2 expression is associated with a decreased baseline rRNA synthesis rate. Decreased baseline rRNA synthesis attenuates p53 stabilization and activation upon RiBi inhibitor treatment, thus promoting chemoresistance. Combinations of RiBi inhibitors and venetoclax with drugs activating p53, irrespective of RiBi inhibition (such as direct MDM2 inhibitors or etoposide), overcome BCL-2 induced resistance *in vitro* and *in vivo* by boosting p53 stabilization levels. We then evaluated the ribosome biogenesis rate in histological sections of DLBCLs from two independent cohorts. For this purpose, we quantified by image analysis the distribution of nucleoli, which are the nuclear structures where rRNA is synthesized, after selective staining with silver. Well-established evidence indicates that the quantitative distribution of nucleoli within the cell nucleus, defined as the nucleolar area, is directly and strictly related to the rRNA transcription rate.^29^ We observed that in DLBCL, the nucleolar area value and ribosome biogenesis rate were highly variable. We demonstrated that a low nucleolar area value, indicative of a low ribosome biogenesis rate, was associated with increased BCL-2 mRNA expression levels and was correlated with poor outcomes following first-line chemoimmunotherapy.

These findings uncover alternative mechanisms of BCL-2 mediated chemoresistance, beyond its known anti-apoptotic activity, providing a rationale for novel combination strategies for BCL-2 positive DLBCL.

## Results

### BCL-2 overexpression promotes resistance to inhibition of ribosome biogenesis in DLBCL cell lines

To explore the therapeutic potential of RiBi inhibition in DLBCL and to identify mechanisms of resistance, we first tested the anti-proliferative activity of CHOP chemotherapy, single agent doxorubicin (Doxo) and Act D, a well-characterized RiBi inhibitor, in a panel of 12 DLBCL cell lines. We then investigated and compared the effects of these agents on rRNA synthesis rate and caspase-induced apoptosis.

**Figure 1:**
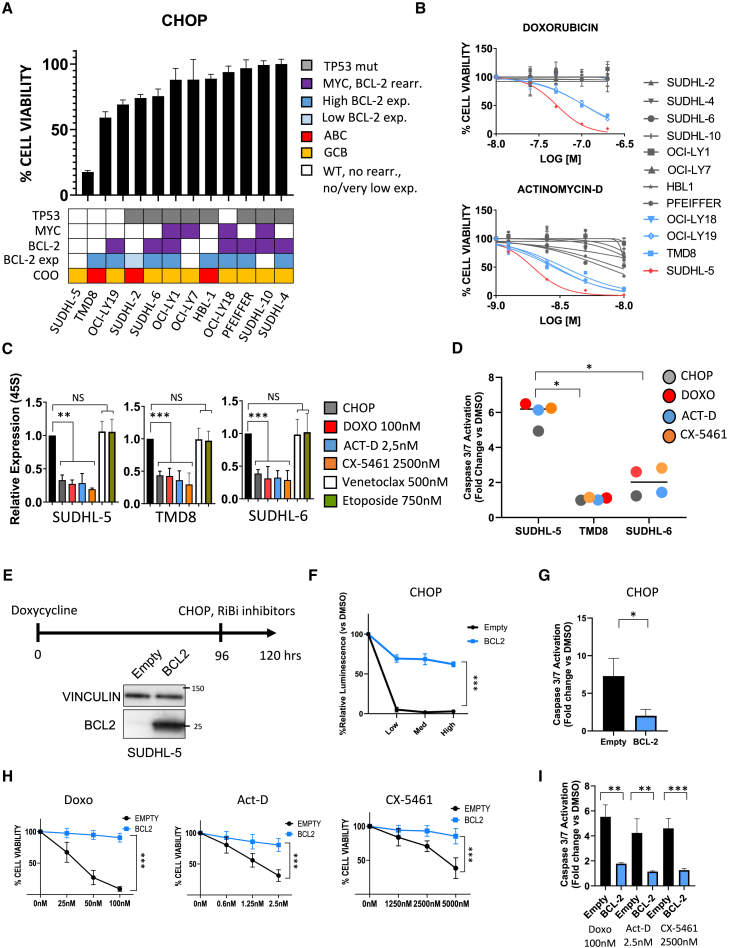
BCL-2 overexpression promotes resistance to inhibition of ribosome biogenesis in diffuse large B cell lymphoma cell lines (A) Bar graph showing antiproliferative effects of CHOP chemotherapy on 12 diffuse large B cell lymphoma (DLBCL) cell lines, measured by CellTiter-Glo assay (CTG). Cells were incubated for 24 h with vincristine 0.37 nM, doxorubicin 50 nM, acrolein 1.5 μM, and methylprednisolone 12.5 μM. Error bars represent standard deviation (SD) of triplicate experiments (*n* = 3). The heatmap shown below indicates the *TP53, MYC* and *BCL-2* status and the cell of origin (COO). (B) CTG assay showing the effects of increasing doses of doxorubicin and actinomycin D on cell viability in 12 DLBCL cell lines after 24 h: in gray, *TP53* mutant cell lines; in blue, BCL-2 positive/*TP53* WT cell lines; in red, the BCL-2 negative/*TP53* WT SUDHL-5 cell line. Error bars represent SD of triplicate experiments (*n* = 3). (C) qPCR analysis of 45S rRNA expression in three DLBCL cell lines incubated for 6 h with the indicated treatments: CHOP (vincristine 0.75 nM, doxorubicin 100 nM, acrolein 3 μM, and methylprednisolone 25 μM) and single agents RiBi inhibitors. Error bars represent SD of five independent experiments (*n* = 5). Student’s t test: ∗∗*p* < 0.01, ∗∗∗*p* < 0.005. (D) Scatterplot representing the effects of 12 h incubation with CHOP and single agent RiBi inhibitors on caspase 3/7 activation (as measured by Caspase-Glo assay) in SUDHL-5, TMD8, and SUDHL-6 cell lines. Each dot represents the mean of triplicate experiments (*n* = 3). Student’s t test: ∗*p* < 0.05. (E) Scheme of BCL-2 overexpression experiments. Western blot shows BCL-2 protein levels in SUDHL-5 cells carrying the empty vector (empty) or the BCL-2 TET-ON inducible system (BCL-2) after 96 h of incubation with 1 μg/ml doxycycline. (F) CTG assay showing the cytotoxic effects at 24 h of three doses of CHOP (vincristine 1.5-0.75-0.37 nM, doxorubicin 200-100-50 nM, acrolein 6-3-1.5 μM, and methylprednisolone 50-25-12.5 μM) in SUDHL-5 cells in the presence or absence of BCL-2. Error bars represent SD of five independent experiments (*n* = 5). Student’s t test: ∗∗∗*p* < 0.005. (G) Caspase-Glo assay showing levels of caspase 3/7 activation in SUDHL-5 cells treated for 12 h with CHOP (vincristine 0.75 nM, doxorubicin 100 nM, acrolein 3 μM, and methylprednisolone 25 μM) in the presence or absence of BCL-2. Error bars represent SD of triplicate experiments (*n* = 3). Student’s t test: ∗*p* < 0.05. (H and I) Effects of 24 h treatment with single agent RiBi inhibitors on cell viability (H) and apoptosis (I) in the presence or absence of BCL-2. Error bars represent SD of five independent experiments (*n* = 5). Student’s t test: ∗∗*p* < 0.01, ∗∗∗*p* < 0.005.

CHOP was modeled *in vitro* by combining Doxo, acrolein (a metabolite of cyclophosphamide known to inhibit rRNA synthesis),^3^ vincristine, and methylprednisolone. CHOP chemotherapy exerted cytotoxic effects in a dose-dependent manner (Figure S1A). As expected, *TP53* mutant cell lines were less sensitive, irrespective of *MYC* and *BCL-2* status (Figures 1A and S1B–S1D). Among *TP53*-WT cell lines, the BCL-2 negative cell line SUDHL-5 was the most sensitive, whereas BCL-2 positive cell lines were less susceptible to the cytotoxic effects of CHOP chemotherapy (Figures 1A and S1A–S1D). Similar results were observed with single agent Doxo and Act D (Figures 1B and S1E).

As shown in Figure 1C, CHOP chemotherapy and single agent Doxo inhibited 45S rRNA synthesis with similar potency, indicating that Doxo was the main effector mediating the inhibition of rRNA synthesis in the CHOP regimen. Similar results were observed with low-dose Act D and CX-5461, a pleiotropic drug initially known only as an RNA polymerase I inhibitor,^30^ which was used as a positive control for rRNA synthesis inhibition. In contrast, the BH3-mimetic venetoclax and the chemotherapeutic drug etoposide, used as negative controls, did not exert any effect on rRNA synthesis (Figure 1C). Notably, while 45S rRNA synthesis was inhibited by RiBi inhibitors to a similar extent irrespective of *TP53* and BCL-2 status, rRNA synthesis blockade translated into a significant induction of apoptosis only in the *TP53* WT BCL-2 negative SUDHL-5 cell line. Similar behavior was observed with CHOP, single agent Doxo, Act D, and CX-5461, indicating a class effect (Figures 1D and S1F). Cell cycle changes induced by RiBi inhibitors were more pronounced in the BCL-2 negative SUDHL-5 cell line compared to BCL-2 positive TMD8 cells. No effects on the cell cycle were observed in *TP53* mutant SUDHL-6 cells (Figure S1G).

Following these observations, to investigate the role of BCL-2 in mediating resistance to RiBi inhibitors, we used a TET-on inducible system to overexpress BCL-2 (coding sequence) in the RiBi-inhibitors sensitive *TP53*-WT BCL-2 negative SUDHL-5 cell line. Cells were preincubated with doxycycline for 96 h and then treated with DMSO, CHOP, Doxo, Act D, or CX5461 for an additional 24 h (Figure 1E). BCL-2 overexpression significantly inhibited the cytotoxic activity of CHOP, single agent Doxo, Act D, and CX-5461, by attenuating caspase 3/7 cleavage (Figures 1F–1I). In line with these findings, BCL-2 silencing using two different short hairpin RNAs (shRNAs) sensitized BCL-2 positive cell lines (OCI-LY19 and TMD8) to the antiproliferative activity of RiBi inhibitors (Figures S2A–S2C).

### Enforced BCL-2 expression is associated with a reduced rRNA synthesis rate in DLBCL cell lines

**Figure 2:**
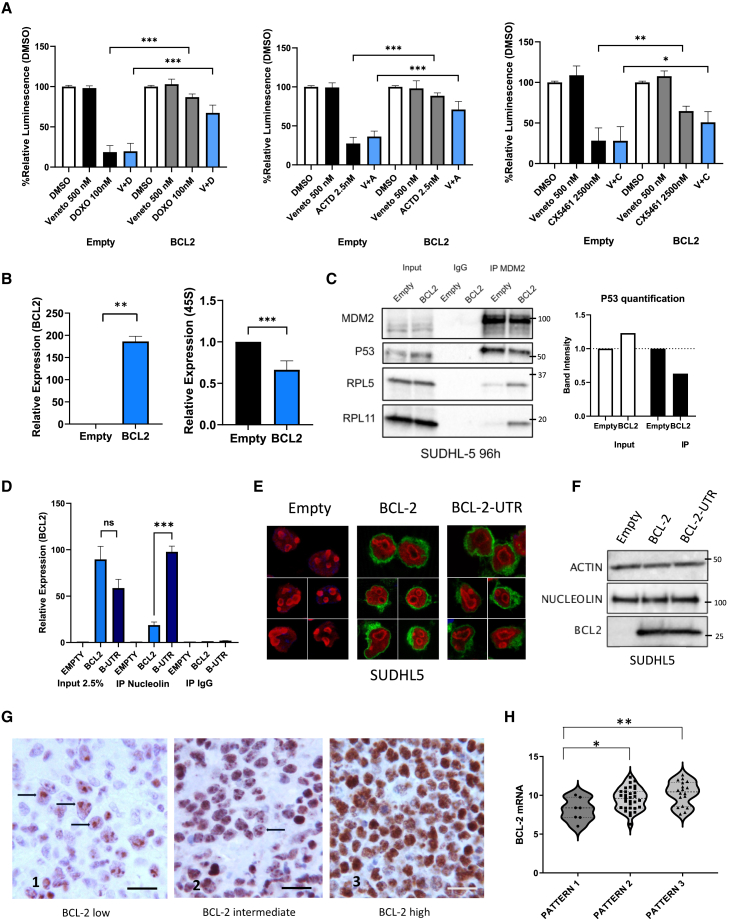
Enforced BCL-2 expression is associated with a reduced rRNA synthesis rate in DLBCL cell lines (A) Bar graph showing the cytotoxic effects of the indicated treatments in the absence or presence of BCL-2. Cells were preincubated with doxycycline 1 μg/ml for 96 h and then treated for 24 h. Error bars represent standard deviation (SD) of five experiments. Student’s t test: ∗*p* < 0.05, ∗∗*p* < 0.01, ∗∗∗*p* < 0.005. V, venetoclax; D, doxorubicin; A, actinomycin D; and C, CX-5461. (B) qPCR analysis showing the levels of *BCL-2* mRNA and 45S rRNA in SUDHL-5 cells transduced with an Empty vector or a BCL-2 doxycycline (TET-ON) inducible system, treated with doxycycline for 96 h. Error bars represent SD of five independent experiments (*n* = 5). Student’s t test: ∗∗*p* < 0.01. (C) Co-immunoprecipitation (coIP) experiment showing the effects of BCL-2 overexpression on MDM2-p53-RPs binding in SUDHL-5 cells in the presence or absence of BCL-2. Cells were incubated with doxycycline for 96 h before harvesting for coIP analysis. Left: western blot analyses showing the expression levels of the indicated proteins in the initial lysates (INPUT) and the levels of MDM2-P53 or MDM2-RPs complexes immunoprecipitated with an MDM2 antibody. Right: protein quantification of the coIP experiment performed using the ImageJ software. (D) Immunoprecipitation-qPCR (IP-qPCR) analysis demonstrating the interaction between nucleolin and BCL-2 mRNA in SUDHL-5 cells. The experiment was conducted in the presence or absence of the BCL-2 coding sequence (BCL2) or the BCL2 3′ untranslated region (B-UTR). Cells were treated with doxycycline for 96 h prior to nucleolin immunoprecipitation. qPCR analysis quantified the BCL2 mRNA levels in the initial sample (INPUT) and in the nucleolin-BCL2 mRNA complexes immunoprecipitated using a nucleolin-specific antibody (IP nucleolin). Error bars represent SD of triplicate experiments (*n* = 3). Student’s t test: ∗∗∗*p* < 0.005. (E) Representative immunofluorescence images of SUDHL-5 cells showing the expression patterns of nucleolin (red) and BCL-2 (green) proteins. Cells were transduced with an empty vector (empty), BCL-2 (BCL-2), or the BCL-2 3′ untranslated region (B-UTR) using a TET-ON system and were incubated with doxycycline for 96 h. (F) Representative western blots showing total protein expression levels of nucleolin and BCL-2, under the same experimental conditions as described in Figure 2E. (G) Immunohistochemistry representative images showing three different patterns of nucleolin distribution in primary FFPE DLBCL tissues, depending on BCL-2 expression levels. Bar, 10 μm. (H) Violin plot showing the correlation between the three patterns of nucleolin distribution and BCL-2 mRNA levels, as measured by T-GEP. Student’s t test: ∗*p* < 0.05, ∗∗*p* < 0.01.

To investigate whether BH3-mimetics could overcome BCL-2 mediated resistance, we evaluated the effects of venetoclax in combination with Doxo, Act D, and CX-5461 in SUDHL-5 cells in the absence or presence of BCL-2. Importantly, as shown in Figure 2A, the addition of venetoclax did not fully reverse BCL-2-mediated resistance in this model. In fact, although venetoclax (at relatively high doses, 500 nM) showed some additive effects in combination with RiBi inhibitors (Doxo, Act D, or CX-5461), these combinations were significantly less effective in the presence of BCL-2 and were unable to completely abrogate BCL-2-mediated pro-survival activity.

To define the functional implications of these findings and to evaluate possible interactions between BCL-2 and RiBi, we first investigated the effects of enforced BCL-2 overexpression on RiBi rate. Notably, enforced BCL-2 expression resulted in decreased 45S rRNA synthesis, indicative of a reduced RiBi rate (Figure 2B). In line with this observation, BCL-2 silencing induced an increase in 45S rRNA synthesis (Figure S2D). To investigate the effects of BCL-2 overexpression on the RP-MDM2-p53 axis, we performed MDM2 co-immunoprecipitation experiments in SUDHL-5 cells in the presence or absence of BCL-2. In line with the inhibition of the 45S rRNA synthesis rate, BCL-2 overexpression resulted in increased binding of RPs RPL11 and RPL5 to MDM2 (Figure 2C), which was associated with a reduced p53-MDM2 interaction, leading to a modest upregulation of p53 protein levels that translated in a mild increase of the G1 fraction of the cell cycle in the absence of cell death, and in a slightly decreased proliferation rate (Figures S3A–S3E). Similar inhibition of 45S rRNA synthesis upon BCL-2 overexpression was observed in *TP53* mutant OCI-LY7 cells, with no signs of p53 stabilization and minor effects on cell proliferation (Figures S3F–S3H). Taken together, these data indicate that BCL-2 overexpression leads to a reduced rRNA synthesis rate irrespective of the *TP53* mutational status in DLBCL cell lines; however, in a *TP53* WT background (SUDHL-5 cell line), these changes translate into weak activation of the RP-MDM2-P53 axis, leading to minor changes in cell proliferation and cell cycle.

Following these observations, we next investigated the effects of BCL-2 overexpression on biomarkers of nucleolar stress, considering the possibility that the reduced rRNA synthesis might be due to a nucleolar stress induced by BCL-2 overexpression. Under normal conditions, the nucleolar protein nucleolin is located in the nucleolus, where it is necessary for rRNA transcription^31^^,^^32^; however following nucleolar stress, nucleolin translocates to the nucleoplasm. On the other hand, BCL-2 mRNA may directly interact with nucleolin through its 3′ untranslated region (UTR).^33^^,^^34^^,^^35^ In this context, in order to dissect the mechanisms underlying BCL-2-induced inhibition of rRNA synthesis, we used a second BCL-2 construct comprising the BCL2 coding sequence and the regulatory 3′UTR region. As shown in Figure 2D and in line with the literature, we confirmed that the full BCL-2-UTR construct directly interacted with nucleolin to a significantly higher extent compared to the BCL-2-coding sequence construct (Figures 2D and S3I). However, despite this difference, the observed phenotype was similar, as both constructs inhibited rRNA synthesis and attenuated the effects of CHOP in DLBCL cells in a similar way (Figures S3J and S3K). Immunofluorescence studies revealed that overexpression of both BCL-2 constructs induced phenotypic changes indicative of nucleolar stress in SUDHL-5 cells, as demonstrated by nucleolin delocalization from the nucleolar body to the nucleoplasm, with no effects on protein abundance (Figures 2E and 2F). These findings were confirmed by immunohistochemical analysis of 56 formalin-fixed paraffin-embedded (FFPE) DLBCL tissue samples from cohort 1 (see detailed description of patient cohorts in the STAR Methods section). By investigating the patterns of nucleolin expression with respect to *BCL-2* mRNA levels measured using targeted gene expression profiling (T-GEP), we observed a significant association between *BCL-2* mRNA levels and nucleolin delocalization in the nucleoplasm (Figure 2G): in samples with low *BCL-2* mRNA expression, nucleolin appeared to be exclusively located in the nucleolus (pattern 1), whereas in samples with increased levels of *BCL-2* mRNA, nucleolin appeared to be progressively distributed in the whole nucleus (patterns 2 and 3) (Figure 2H). Therefore, taken together, these data indicate that overexpression of *BCL-2* mRNA reduces rRNA synthesis inducing a nucleolar stress that is, in turn, responsible for nucleolin translocation.^36^

### BCL-2 overexpression attenuates p53 stabilization and activation following RiBi inhibitors treatment

**Figure 3:**
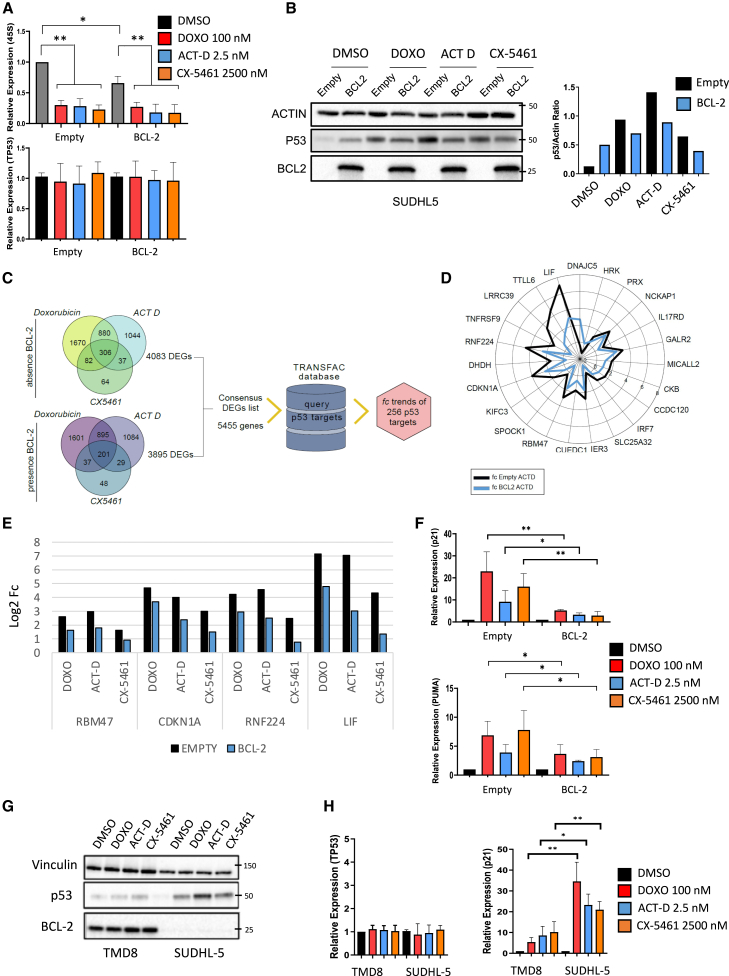
BCL-2 overexpression attenuates p53 stabilization and activation following RiBi inhibitors treatment (A) qPCR analysis showing the expression levels of 45S rRNA and *TP53* mRNA in SUDHL-5 cells incubated for 24 h with the indicated treatments in the presence or absence of BCL-2. Error bars represent standard deviation (SD) of triplicate experiments (*n* = 3). Student’s t test: ∗*p* < 0.05,∗∗*p* < 0.01. (B) Representative immunoblots showing the effect of single agents RiBi inhibitors on P53 protein levels in SUDHL-5 cells in the presence or absence of BCL-2 (same conditions as Figure 3A). Protein quantification (right) was performed using ImageJ software. (C) RNA-seq experiment performed in SUDHL-5 cells incubated with RiBi inhibitors for 6 h in the presence or absence of BCL-2 (after 96 h induction with doxycycline). The experiment was performed in triplicate (*n* = 3). Differentially expressed genes (DEGs) were reported in the Venn diagrams. A consensus DEGs list was extracted and matched with the TRANSFAC database to focus on differentially regulated p53 target genes and explore their expression in the absence or presence of BCL-2 following treatment with RiBi inhibitors. (D) Radar chart displaying fold changes of significantly regulated p53 target genes identified in SUDHL-5 cells treated with actinomycin D in the presence (blue line) and absence (black line) of BCL-2. (E) Bar graph showing fold change of common p53 target genes identified in SUDHL-5 cells following treatment with different RiBi inhibitors in the presence or absence of BCL-2. (F) qPCR analysis showing expression of representative p53 targets, *CDKN1A* (p21) and *PUMA*, in SUDHL-5 cells incubated 24 h with doxorubicin (100 nM), actinomycin D (2.5 nM), and CX-5461 (2,500 nM) in the presence or absence of BCL-2. Error bars represent SD of 5 independent experiments (*n* = 5). Student’s t test: ∗*p* < 0.05, ∗∗*p* < 0.01. (G) Representative western blots showing the effect of 24 h treatment with doxorubicin (100 nM), actinomycin D (2.5 nM), and CX-5461 (2,500 nM) on p53 protein levels in TMD8 (BCL-2 positive) and SUDHL-5 (BCL-2 negative) cells. (H) qPCR analysis showing mRNA expression levels of *TP53* and *CDKN1A* (p21) in TMD8 and SUDHL-5 cell lines incubated for 24 h with doxorubicin (100 nM), actinomycin D (2.5 nM), or CX-5461 (2,500 nM). Error bars represent SD of 5 independent experiments (*n* = 5). Student’s t test: ∗*p* < 0.05, ∗∗*p* < 0.01.

Since the baseline level of rRNA synthesis (which is indicative of baseline ribosome biogenesis rate) correlates with the efficacy of RiBi inhibitors by regulating the amount of p53 stabilized upon drug-induced nucleolar stress,^11^ we next investigated the dynamic effects of RiBi inhibitor treatment on the p53 axis in the absence or presence of BCL-2, hypothesizing that a reduced baseline rRNA synthesis rate induced by BCL-2 would determine reduced levels of stabilized p53 following RiBi inhibitors exposure. As shown in Figure 3A, RiBi inhibitors (Doxo, Act D, and CX-5461) significantly downregulated rRNA synthesis irrespective of BCL-2 expression levels, in the absence of significant *TP53* mRNA induction. However, in line with our hypothesis, upon drug-induced inhibition of rRNA synthesis, the amount of stabilized p53 was decreased in the presence of BCL-2 (Figure 3B). To further investigate the functional consequences of these findings, we performed RNA sequencing (RNA-seq) analyses of SUDHL-5 cells treated with RiBi inhibitors (Doxo, Act D, and CX5461) in the absence or presence of BCL-2.

Principal-component analysis (PCA) revealed that BCL-2 expression was the main driver of sample segregation, followed by exposure to RiBi inhibitors (Figure S4A). Differentially expressed genes (DEGs) were identified by comparing different RiBi inhibitors (Doxo, Act D, CX-5461) versus control samples (DMSO) in the presence or absence of BCL-2 to identify candidate genes reflecting distinctive profiles. In the absence of BCL-2 (empty vector), 3,890, 3,170, and 697 DEGs were identified respectively in Doxo-, Act D-, and CX-5461-treated SUDHL-5 cells; in the presence of BCL-2, 3,641, 3,118, and 441 DEGs were identified in Doxo, Act D, and CX-5461 respectively.

Detailed results of the differential expression analysis for all possible pairwise comparisons are available in the supplementary material (Data S1). Functional enrichment analysis was performed using DEGs identified in the six pairwise comparisons using clusterProfiler. According to Kyoto Encyclopedia of Gene and Genome (KEGG) pathway enrichment analysis, several pathways, including p53 signaling and cell cycle, were enriched upon treatment with RiBi inhibitors either in the absence or presence of BCL-2 (Figures S4B and S4C). However, the apoptotic pathway was enriched only in the absence of BCL-2, with 37 genes exclusively regulated in the empty vector-transduced SUDHL-5 cells (Figure S4C, and Data S2). These genes belonged to the p53 signaling, tumor necrosis factor, mitogen-activated protein kinase, and calcium signaling networks; 9 of the 37 genes were direct p53 targets. To further investigate dynamic changes in the regulation of the p53 network, which could be missed by pathway enrichment analysis, we created a DEGs consensus list and queried the TRANSFAC database (a public dataset containing a list of direct p53 targets); we obtained 256 direct p53 targets differentially expressed in the six pairwise comparisons (Figure 3C). In order to capture biologically relevant differences in the degree of activation of p53 targets in response to RiBi inhibitors treatment according to the absence or presence of BCL-2, we applied a 2-step filter: we considered only those genes with log2 fold change (fc) vs. DMSO ≥1 and with ≥0.58 log2 fc difference between absence and presence of BCL-2 in each treatment arm (Doxo, Act D, and CX-5461) (|Δ*fc*|≥0.58log2 [Δ*fc* = fc_empty_−fc_BCL-2_]). As shown in the representative radar chart depicted in Figure 3D, the induction of p53 targets following Act D treatment was significantly attenuated in the presence of BCL-2. Similar results were obtained with Doxo and CX-5461 (Figures 3E, S4D, and S4E).

Significant attenuation of p53 targets induction (*CDKN1A* [p21] and *PUMA*) was confirmed by qPCR in SUDHL-5 cells following BCL-2 overexpression (Figure 3F).

In line with these findings and with the data shown in Figure 1, the *TP53* WT BCL-2 positive TMD8 cell line was characterized by attenuated p53 stabilization and target activation upon RiBi inhibitors treatment, compared to the BCL-2 negative SUDHL-5 cell line (Figures 3G, 3H, and S4F). Notably, according to our previous findings, the BCL-2 negative SUDHL-5 cell line displayed higher baseline 45S rRNA levels compared to *TP53* WT BCL-2 positive DLBCL cell lines (Figure S4G).

### MDM2 inhibitors in combination with venetoclax overcome BCL-2-mediated resistance to RiBi inhibitors *in vitro* and *in vivo*

Having demonstrated that BCL-2 attenuates p53-mediated responses to RiBi inhibitors, we investigated whether boosting p53 activation by inhibiting MDM2-p53 interactions with small molecule MDM2 inhibitors (MDM2i) would be an effective strategy to overcome BCL-2 mediated resistance.

**Figure 4:**
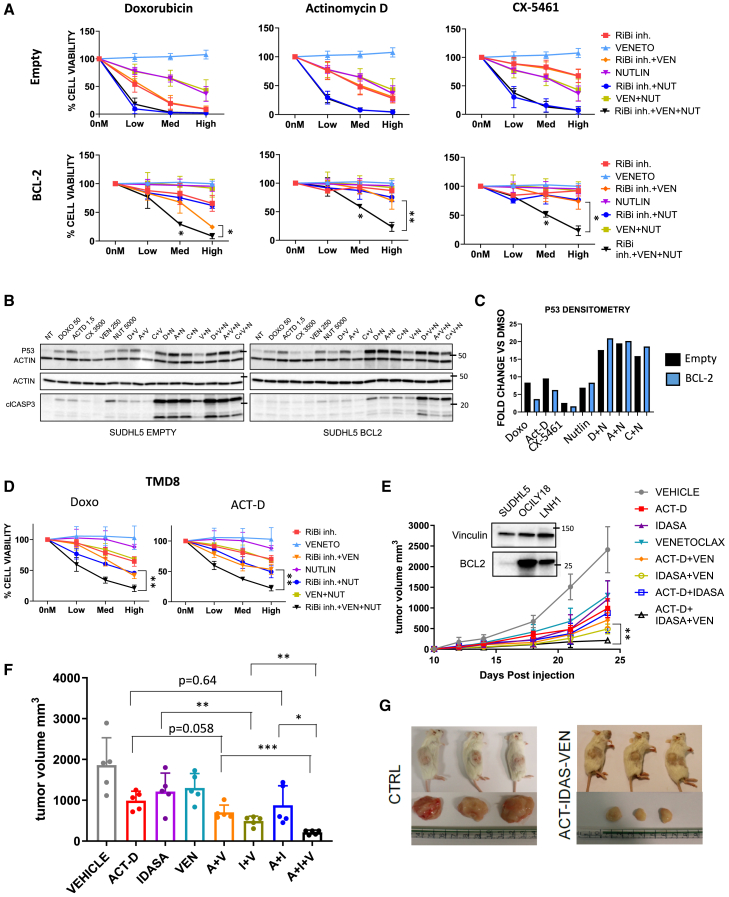
MDM2 inhibitors in combination with venetoclax overcome BCL-2-mediated resistance to RiBi inhibitors *in vitro* and *in vivo* (A) CTG assay showing cell viability of SUDHL-5 cells treated for 24 h with RiBi inhibitors (doxorubicin 25, 50, 100 nM; actinomycin D 0.65, 1.25, 2.5 nM; and CX-5461 625, 1250, 2,500 nM), venetoclax (125, 250, 500 nM), and with the MDM2i nutlin-3A (1,250, 2,500, 5,000 nM) as single agents or in different combinations, in the presence or absence of BCL-2. Error bars represent standard deviation (SD) of triplicate experiments (*n* = 3). Student’s t test: ∗*p* < 0.05, ∗∗*p* < 0.01. (B) Representative western blot showing the effects of the indicated treatments on p53 protein abundance and caspase 3 cleavage in SUDHL-5 cells in the presence or absence of BCL-2. Cells were pre-treated with doxycycline for 96 h and then incubated with doxorubicin 100 nM, actinomycin D (2.5 nM), CX-5461 (2,500 nM), venetoclax (500 nM), nutlin-3A (2,500 nM), and the indicated combinations for 24 h. (C) Bar graph depicting p53 densitometry analysis of the immunoblot shown in Figure 4B. Densitometry analyses were performed using ImageJ software. (D) CTG assay showing cell viability of TMD8 cells treated for 24 h with RiBi inhibitors (doxorubicin 25, 50, 100 nM; actinomycin D 0.65, 1.25, 2.5 nM), venetoclax (125, 250, 500 nM), and the MDM2i nutlin-3A (1,250, 2,500, 5,000 nM) as single agents or in combination. Error bars represent SD of triplicate experiments (*n* = 3). Student’s t test: ∗∗*p* < 0.01. (E) *In vivo* combination experiment in a subcutaneous TP53 WT/BCL-2 positive DLBCL PDX model (LNH1). NSG mice were treated with vehicle, 0.04 mg/kg actinomycin D, 50 mg/kg venetoclax, and 100 mg/kg idasanutlin (MDM2i) as single agents or in combination. Tumor volume was measured using a caliper. Error bars represent SD of five mice (*n* = 5). The representative western blot shows BCL-2 baseline protein level in the LNH1 PDX, as well as in SUDHL-5 and OCILY-18 cell lines (used as negative and positive controls, respectively). (F) Bar graph showing tumor volume at day 24 (same experiment Figure 4E). Error bars represent SD of five mice (*n* = 5). Student’s t test: ∗*p* < 0.05, ∗∗*p* < 0.01, ∗∗∗*p* < 0.005. (G) Representative image of three mice and their tumors at day 24 comparing vehicle-treated versus triple combination treated (actinomycin D, venetoclax, and idasanutlin).

According to the underlying hypothesis, a triple combination of RiBi inhibitors (Doxo, Act D, or CX5461), nutlin 3A (MDM2i), and venetoclax was able to fully reverse BCL-2-mediated resistance in SUDHL-5 cells (Figure 4A). Interestingly, the addition of nutlin 3A to RiBi inhibitors resulted in enhanced p53 induction (which was slightly more evident after enforced BCL-2 expression) (Figures 4B and 4C), indicating a synergistic effect of MDM2i and RiBi inhibitors on p53 stabilization. In line with the restored p53 stabilization levels, a detectable caspase 3 cleavage was clearly observed with the triple combination (RiBi inhibitors + venetoclax + nutlin) in BCL-2 positive SUDHL-5 cells (Figure 4B).

According to this p53-dependent mechanism of action, the triple combination of RiBi inhibitors (Doxo or Act D), nutlin 3A, and venetoclax showed enhanced efficacy, with increased p53 induction and enhanced apoptosis also in the *TP53* WT TMD8 cell line (Figures 4D and S5A), but not in *TP53* mutant SUDHL6 cells (Figure S5B).

To further corroborate these findings, we assessed whether similar results could be obtained using different drugs inducing p53-mediated responses via RiBi-independent mechanisms, such as etoposide.^37^ In line with this hypothesis, the addition of etoposide to a combination of RiBi inhibitors and venetoclax reversed BCL-2 mediated resistance in SUDHL-5 cells (Figures S5C and S5D).

We next investigated the efficacy of triple RiBi-BCL2-MDM2 inhibition *in vivo* in a subcutaneous patient-derived xenograft (PDX) model of DLBCL.^38^ This PDX, obtained from a nodal biopsy performed at relapse in a DLBCL patient treated with first-line R-CHOP chemoimmunotherapy, was characterized by high BCL-2 expression (Figure 4E) and a lack of genomic alterations in *TP53*. After PDX engraftment, NOD.Cg-PrkdcSCIDIl2rgtm1Wjl/SzJ (NSG) mice were treated with Act D, venetoclax, and idasanutlin (MDM2i) as monotherapy or in combination with different regimens (Act D + venetoclax, idasanutlin + venetoclax, Act D + idasanutlin, and Act D + idasanutlin + venetoclax) for 14 days (Figures 4E–4G). In line with our *in vitro* data, the triple combination of Act D, venetoclax, and idasanutlin exerted synergistic effects in this model, significantly reducing tumor growth compared to doublet combination (Act D + venetoclax, idasanutlin + venetoclax, Act D + idasanutlin) (Figures 4E–4G). We did not observe significant weight loss in mice treated with Act D, idasanutlin, and venetoclax, as single agent or in combination (Figures S5E and S5F).

### BCL-2 overexpression is associated with decreased nucleolar area and adverse outcome in DLBCL

In order to investigate the relationship between known prognostic factors, *BCL-2* mRNA levels, RiBi rate and disease outcome, we profiled an exploratory cohort of 83 DLBCL patients treated with anthracycline-based first-line chemoimmunotherapy (cohort 1, see STAR Methods), with T-GEP (for the assessment of the COO, and measurement of *BCL-2* and *MYC* mRNA levels) and with silver staining of histological sections to determine the nucleolar area. In fact, well-established evidence indicates that the nucleolar area is directly and strictly related to the rRNA transcription.^29^

**Figure 5:**
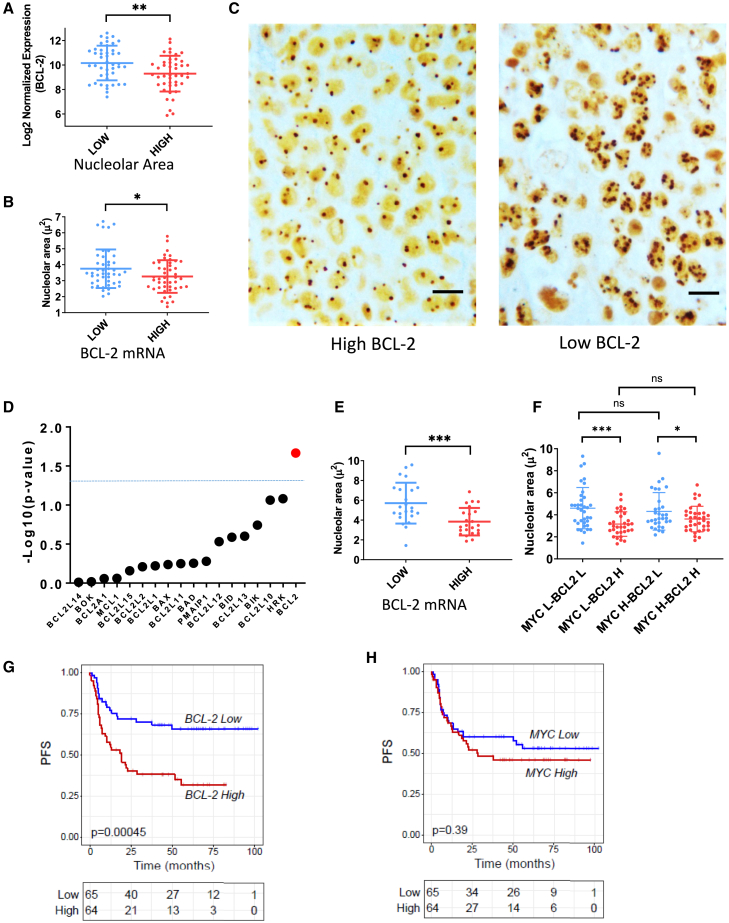
BCL-2 overexpression is associated with decreased nucleolar area and adverse outcome in diffuse large B cell lymphoma (A) Dot plot graph showing *BCL-2* mRNA expression levels (measured by T-GEP) in patient samples (exploratory cohort 1, *n* = 83) with low and high nucleolar area, as determined by quantitative image analysis of silver-stained nucleolar structures. The definition of “high” and “low” nucleolar area was based on the median value of the nucleolar area in the whole cohort. Student’s t test: ∗∗*p* < 0.01. (B) Dot plot graph showing values of nucleolar area in patient samples (exploratory cohort 1, *n* = 83) characterized by low and high BCL-2 mRNA levels. The definition of “high” and “low” BCL-2 mRNA expression was based on the median value of BCL-2 mRNA expression in the whole cohort as determined by T-GEP. Student’s t test: ∗*p* < 0.05. (C) Representative nucleolar silver staining of DLBCL histological sections from two patients with high (left) and low (right) BCL-2 mRNA expression. Scale bar, 10 μm. D) Dot plot graph showing the correlation significance between mRNA levels of several BCL-2 family members and nucleolar area in the exploratory cohort. Among all BCL-2 family members, only BCL-2 mRNA levels show a significantly association with nucleolar size. (E) Dot plot graph showing nucleolar area values in patient samples (validation cohort 2, *n* = 46) characterized by low and high BCL-2 mRNA levels. Student’s t test: ∗∗∗*p* < 0.005. (F) Dot plot graph showing nucleolar area values in patient samples (cohort 1 + cohort 2, *n* = 129) according to *BCL-2* and *MYC* mRNA levels. L indicates “low,” and H indicates “high.” Student’s t test: ∗*p* < 0.05, ∗∗∗*p* < 0.005. (G) Progression-free survival (PFS) curve of the whole cohort (cohort 1 + cohort 2, *n* = 129) according to *BCL-2* mRNA expression levels. *p* value was calculated using the log-rank test. (H) PFS curve of the whole cohort (cohort 1 + cohort 2, *n*= 129) according to *MYC* mRNA expression levels. *p* value was calculated using the log-rank test.

As shown in Table 1, nucleolar area was highly variable from case to case ranging from 1.37 micron^2^ (μ^2^) to 6.71 μ^2^. No significant associations were observed between COO and nucleolar area (Figure S6A). Notably, we observed an inverse relationship between *BCL-2* mRNA levels and nucleolar area, with higher *BCL-2* mRNA levels associated with decreased nucleolar area (Figures 5A–5C). High and low gene expression levels and high and low nucleolar area values were defined based on the respective median values.

**Table 1:** Patient characteristics

	Exploratory cohort	Validation cohort	Combined cohort
N° patients	83	46	129
Age Median, range	67 (21–87)	53 (23–61)	60 (21–87)
COO Hans GC Non GC	37 46	16 30	53 76
COO T-GEP GC ABC Unclassified	62 8 13	23 15 8	85 23 21
AgNOR area (μ^2^) Median, range	3.38 (1.37–6.71)	4.67 (1.44–9.58)	3.55 (1.37–9.58)
aaIPI score 1 2 3	8 50 25	– 36 10	8 86 35
DEXP (IHC) Yes No	21 62	13 33	34 95
FISH status MYC BCL-2 BCL-6 DHIT	5 7 26 2	5 8 17 5	10 15 43 7
DEXP T-GEP Yes No	21 62	14 32	35 94
BCL-2 mRNA Median, range	9.86 (5.88–12.62)	8.99 (6.03–11.68)	9.30 (5.88–12.62)
MYC mRNA Median, range	10.22 (7.51–13.97)	8.98 (7.31–10.77)	9.83 (7.31–13.97)
P53 (IHC) Positive Negative NE	17 53 3	6 38 2	23 101 5

To better investigate the specificity of this observation, we profiled our exploratory cohort with a custom T-GEP panel including all BCL-2 family members (Data S3); of note, *BCL-2* was the only member of the BCL-2 family whose mRNA levels were significantly associated with the nucleolar area values (Figure 5D). Interestingly, among all antiapoptotic BCL-2 family members, only *BCL-2* emerged as a significant predictor of poor outcome, with higher *BCL-2* mRNA levels being correlated with shorter progression-free survival (PFS) following standard chemoimmunotherapy (Figure S6B).

To confirm these findings, we profiled a validation cohort of 46 patients (cohort 2) from the DLCL04 study^39^ using T-GEP and silver staining of nucleolar structures. Again, we observed an inverse relationship between *BCL-2* mRNA levels and nucleolar area (Figures 5E and S6C). Patient characteristics of discovery and validation cohorts are shown in Table 1.

Next, to further dissect the relationship between *MYC*, *BCL-2*, COO, and nucleolar area values, we analyzed a combined cohort of 129 patients (cohort 1 + cohort 2). Again, no correlation was observed between nucleolar area values and COO subtyping, with no significant differences between the activated B cell (ABC) and germinal center (GC) B cell/unclassified subgroups (Figure S6D). In line with our prior findings, we observed a strong inverse correlation between *BCL-2* mRNA levels and nucleolar area in this combined cohort of patients (Figure S6D).

Since MYC is known as a positive regulator of ribosome biogenesis,^40^ we evaluated the relative influence of *MYC* and *BCL-2* mRNA levels on nucleolar area. Importantly, although there was a positive correlation between *MYC* mRNA levels and nucleolar size, which did not reach statistical significance, *BCL-2* mRNA levels were negatively correlated to nucleolar area values irrespective of *MYC* levels: in fact, while increased *BCL-2* mRNA levels were associated with decreased nucleolar size in both *MYC*-low and *MYC*-high subgroups, on the contrary, *MYC* mRNA levels did not correlate with nucleolar area when categorizing based on *BCL-2* mRNA expression levels (Figures 5F and S6E).

Notably, as opposed to *BCL-2*, which was associated with outcome in both cohorts (Figures S7A and S7B), *MYC* mRNA levels were not associated with the outcome in either dataset when analyzed separately (Figures S7C and S7D), and as a whole (*n* = 129 patients) (Figures 5G and 5H).

These data were confirmed *in silico* using two independent publicly available datasets from Lenz et al.^41^ and Sha et al.,^42^ where *BCL-2* mRNA levels outperformed *MYC* as an outcome predictor in patients treated with R-CHOP chemoimmunotherapy (Figures S8A–S8D). To further elucidate the role of MYC in regulating the sensitivity to RiBi inhibition, we used the P-4936 cell line, where MYC expression is under the control of a tetracycline regulated repressible (Tet-OFF) promoter.^43^ We treated P-4936 cells with CHOP or single agent RiBi inhibitors (doxorubicin, Act D, and CX-5461) for 24 h in the presence or absence of MYC. As shown in Figures S8E and S8F, in line with survival analyses depicted in Figure 5, modulation of MYC expression levels had minor effects on CHOP and RiBi inhibitors antiproliferative activity.

### A reduced nucleolar area is an independent predictor of adverse outcome in DLBCL patients treated with standard anthracycline-based chemoimmunotherapy

**Figure 6:**
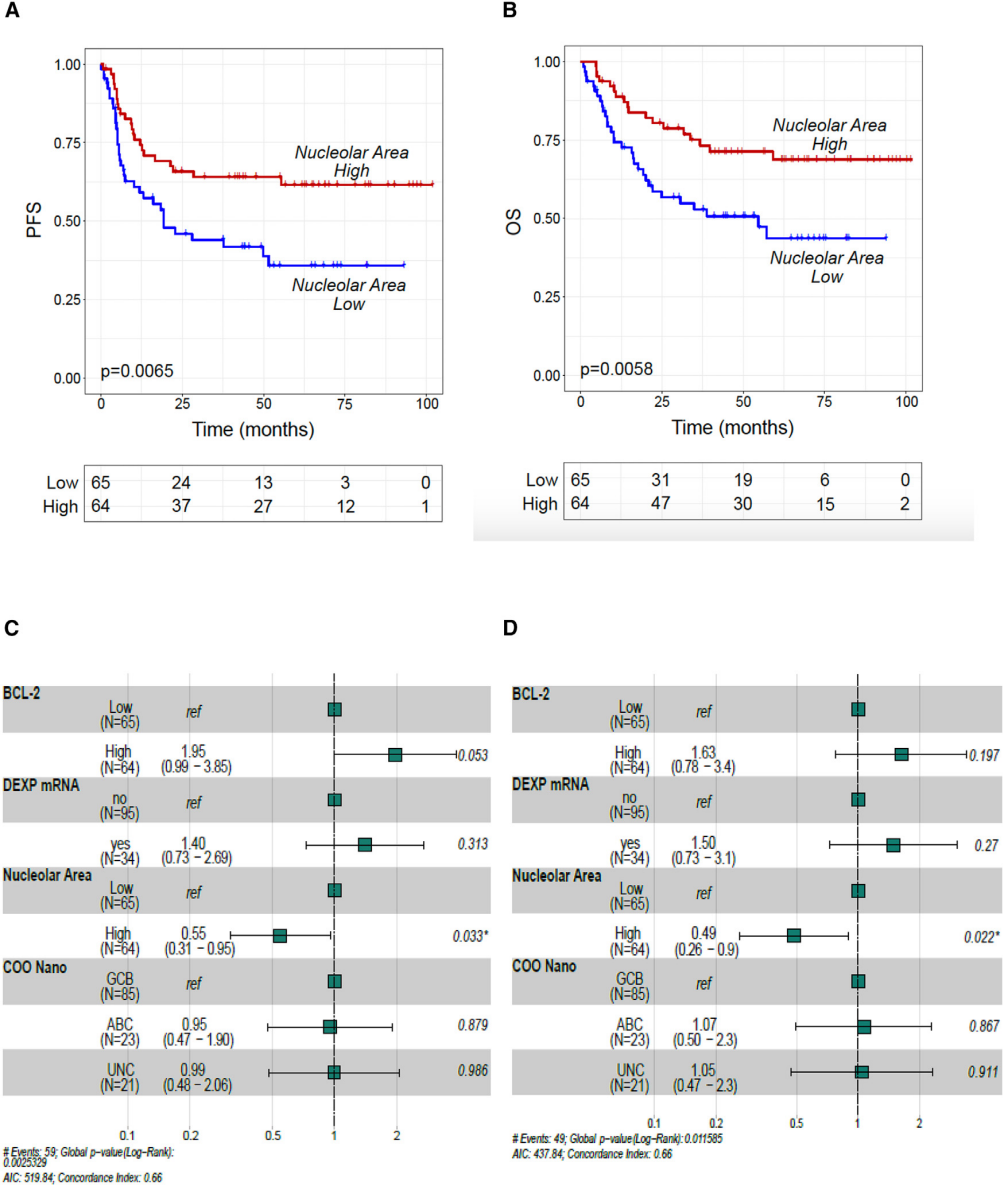
A reduced nucleolar area is an independent predictor of adverse outcome in DLBCL patients treated with standard anthracycline-based chemoimmunotherapy (A) Progression-free survival (PFS) curve of the whole cohort (exploratory + validation, *n* = 129) according to nucleolar size (nucleolar area). The definition of “high” and “low” nucleolar area was based on the median value of the whole cohort. *p* value was calculated using the log-rank test. (B) Overall survival (OS) curve of the whole cohort (exploratory + validation, *n* = 129) according to nucleolar size (nucleolar area). The definition of “high” and “low” nucleolar area was based on the median value of the whole cohort. *p* value was calculated with the log-rank test. (C) Forest plot depicting multivariable analyses for PFS in the whole cohort (exploratory + validation, *n* = 129). (D) Forest plot depicting multivariable analyses for OS in the whole cohort (exploratory + validation, *n* = 129).

Next, given the demonstrated relationship between ribosome biogenesis rate and efficacy of RiBi inhibitors in preclinical models^11^ and considering that inhibition of ribosome biogenesis is a relevant mechanism of action of anthracyclines, we analyzed the correlation between nucleolar area (indicative of ribosome biogenesis rate) and outcome after first-line anthracycline-based therapy in the whole cohort of 129 patients. Nucleolar area was defined as high or low based on the median value. We found that a decreased nucleolar area, indicative of a decreased ribosome biogenesis rate, was associated with poor outcome in terms of PFS and overall survival (OS) (Figures 6A and 6B). Importantly, in multivariate analyses, nucleolar area emerged as the only independent outcome predictor, irrespective of *MYC*, *BCL-2* mRNA levels and COO subtyping (Figures 6C and 6D). Patient’s characteristics were well balanced in the nucleolar area high and low subgroups (Table S1). Finally, these data were confirmed after excluding patients with positive p53 immunostaining (*n* = 23, 17%), which is indicative of the presence of *TP53* mutations^44^ (Figures S9A–S9D).

## Discussion

The R-CHOP chemoimmunotherapy regimen has been the mainstay of DLBCL treatment for decades, and even in the era of novel agents, therapeutic strategies based on small molecule inhibitors, bispecific T cell engagers and immunoconjugates, are under clinical development in combination with R-CHOP as first line therapy. Therefore, a deeper understanding of the mechanisms underlying resistance to R-CHOP chemoimmunotherapy is still timely and relevant.

In the present study we demonstrate that the poor prognosis of DLBCL patients overexpressing BCL-2 is due only in part to the direct antiapoptotic activity mediated by BCL-2, but it is in fact also the consequence of reduced p53 activation upon exposure to chemotherapeutic agents inhibiting ribosome biogenesis, such as anthracyclines. We demonstrate that this reduced sensitivity is due to a downregulation of baseline rRNA synthesis rate associated with BCL-2 overexpression.

Given that BCL-2 is emerging as one of the main prognostic predictors in DLBCL treated with anthracycline-containing regimens, we first investigated the impact of BCL-2 overexpression on the activity of chemotherapeutic drugs inhibiting ribosome biogenesis.

We showed that CHOP treatment induced a potent inhibition of rRNA synthesis in DLBCL cell lines, this activity being mainly attributable to doxorubicin, which mirrored the effects of well-known RiBi inhibitors (such as Act D and CX-5461) on rRNA transcription. Although both doxorubicin and CX-5461 display pleiotropic mechanisms of action, inhibition of rRNA synthesis represents a well-described component of their anticancer activity, and these agents are considered potent RiBi inhibitors. According with the p53-dependent mechanism of action of RiBi inhibitors, pharmacologic inhibition of rRNA synthesis did not translate in significant cytotoxicity in *TP53* mutant cell lines. However, among *TP53* WT cell lines, maximal efficacy was observed in the BCL-2 negative SUDHL-5 cell line, with BCL-2 positive *TP53* WT cell lines being less sensitive. Enforced BCL-2 overexpression rendered SUDHL-5 cells resistant to CHOP and single agent RiBi inhibitors treatment. This resistance was not solely due to the direct antiapoptotic activity of BCL-2, as combined treatment with the BH3-mimetic venetoclax, could not fully reverse BCL-2 induced resistance. Following these observations, we hypothesized that BCL-2 could modulate the activity of RiBi inhibitors with BH3-independent mechanisms.

Indeed, following enforced BCL-2 expression we observed a reduced rRNA synthesis rate in DLBCL cells, which resulted in a modest increase in the fraction of cells in the G1 phase of the cell cycle, in the absence of apoptosis. This is in line with several early studies investigating the antiproliferative effects related to BCL-2 overexpression, showing a delayed G1-S transition in BCL-2 positive cells.^45^

BCL-2 overexpression was associated to nucleolar changes indicative of nucleolar stress, such as the translocation of nucleolin from the nucleolus to the nucleoplasm. Considering the known direct interaction between nucleolin and *BCL-2* mRNA 3′UTR,^33^ we hypothesized that overexpression of *BCL2* mRNA might be responsible for the sequestration of nucleolin in the nucleoplasm, thus determining a reduced availability for the process of ribosome biogenesis. However, similar phenotypes were observed using a BCL-2 construct encoding only for the coding sequence and a full-length BCL-2 construct with UTRs. In fact, although we were able to confirm a direct interaction of the BCL-2-UTR construct with nucleolin, enforced expression of both constructs exerted similar effects in terms of rRNA synthesis inhibition and attenuation of CHOP-induced cytotoxicity. Immunofluorescence experiments carried out in SUDHL-5 cells were recapitulated by immunohistochemistry studies performed on FFPE tissue, confirming that, in the presence of BCL-2, nucleolin translocated from the nucleolus, to the nucleoplasm. These data suggest that BCL-2 overexpression induces a nucleolar stress that is associated, through direct or indirect mechanisms, with a reduced ribosome biogenesis.^36^ In this light, previous studies demonstrated the poor prognostic impact of nucleolin overexpression levels in DLBCL.^46^ Our data indicate that the pattern of nucleolin expression could be a useful biomarker of BCL-2-induced nucleolar stress and chemoresistance in DLBCL.

In line with the known direct relationship between baseline ribosome biogenesis rate and the amount of stabilized p53 upon rRNA synthesis blockade,^11^ BCL-2-associated reduction of baseline rRNA synthesis resulted in attenuated p53 induction after treatment with RiBi inhibitors. RNA-seq experiments performed in SUDHL-5 cells treated with RiBi inhibitors in the presence or absence of BCL-2, indicated that enforced BCL-2 overexpression attenuated the induction of p53 target genes. Taken together these findings indicate that BCL-2 overexpression may induce chemoresistant phenotypes not only by directly inhibiting apoptosis downstream of p53, but also by attenuating p53 activation in response to RiBi inhibitors. Indeed, combinatory strategies aimed at boosting p53 activation, proved to be effective *in vitro* and *in vivo* in BCL-2 positive DLBCL models. More precisely, combinations of RiBi inhibitors with drugs stabilizing p53 in a RiBi-independent manner, such as MDM2i and etoposide, restored efficient stabilization of p53 in BCL-2 overexpressing DLBCL cells. Interestingly the combination of RiBi inhibitors and MDM2i significantly increased p53 levels as compared to either drug used as single agent. This could be explained by the fact that nutlin 3A and RPs (RPL5 and RPL11) bind MDM2 at different sites, thus determining additive effects on p53 induction.^47^

Several prior studies have confirmed the therapeutic synergy and synthetic lethality of MDM2 and BCL-2 inhibitors through p53-dependent mechanisms.^48^^,^^49^ However, the present observations provide a preclinical rationale for combining RiBi inhibitors (including anthracyclines) with MDM2i to maximize p53 induction, which in turn could boost BCL-2 inhibitor-mediated apoptotic responses. Combination treatment with chemotherapeutic agents such as etoposide, that induce activation of the p53 axis irrespective of RiBi inhibition,^37^ had similar effects. In line with this, current evidence suggests an improved outcome with etoposide-containing regimens in double-hit high-grade B cell lymphoma or double-expresser DLBCL.^50^^,^^51^ Results of recent clinical trials in which anthracycline-based chemoimmunotherapy was combined with BCL-2 inhibitors further corroborate our findings: in fact, combinatory strategies based on the addition of venetoclax to R-CHOP produced suboptimal results in DLBCL, failing to provide a significant clinical benefit, as increased toxicities were not counterbalanced by increased effectiveness.^27^^,^^28^ These findings are consistent with a model in which restored p53-mediated responses by RiBi inhibitors-MDM2i or RiBi inhibitors-etoposide combinations could play a crucial role for the induction of synthetic lethality with BH3-mimetics in BCL-2 positive DLBCL.

To determine the clinical implications of our findings, we next investigated whether differences in baseline RiBi rate may influence clinical outcomes in DLBCL patients treated with R-CHOP chemoimmunotherapy. To the best of our knowledge, the relationship between baseline RiBi rate, established biomarkers of DLBCL outcome and the efficacy of CHOP chemotherapy has never been investigated. In line with the observed effects of BCL-2 overexpression on rRNA synthesis rate, we confirmed an inverse correlation between *BCL-2* mRNA levels and nucleolar area in two independent cohorts of DLBCL patients treated with anthracycline-based chemoimmunotherapy. The observed negative correlation was specific, since among all BCL-2 family members, only BCL-2 correlated with nucleolar area. Furthermore, nucleolar area was an independent prognostic predictor in multivariate analysis, outperforming *BCL-2* mRNA levels. Taken together, these data demonstrate a previously unrecognized link between BCL-2 and ribosome biogenesis, suggesting that the adverse prognostic influence related to BCL-2 overexpression could be due, in relevant part, to a reduced ribosome biogenesis rate associated with BCL-2 expression, which in turn impairs p53-mediated response to chemotherapy. In this light, nucleolar area should be considered as an additional prognostic factor in DLBCL, with potential clinical relevance.

Modulation of MYC levels using a TET-OFF system did not significantly affect sensitivity to CHOP or RiBi inhibitors *in vitro*, and in line with this *MYC* mRNA levels were not associated with outcome in survival analyses. These data provide a possible mechanistic explanation to the growing body of evidence suggesting a dominant prognostic role of BCL-2 overexpression and a context-dependent role of MYC in DLBCL treated with R-CHOP.^18^^,^^20^^,^^21^ On the other hand, it is well known that MYC is a master regulator of cancer cell proliferation, metabolism and immune evasion, with pleiotropic effects on cell cycle, DNA repair, and multiple metabolic pathways.^52^^,^^53^^,^^54^ It is possible that the aforementioned functions of MYC could synergize with the effects of a “second hit” (BCL-2 overexpression and/or TP53 mutation) affecting sensitivity to RiBi inhibition, thus contributing to determine the clinical aggressiveness and chemoresistant phenotypes observed in MYC/BCL-2 double-expresser lymphoma.

In summary, this study uncovered alternative mechanisms of chemoresistance related to BCL-2 overexpression beyond its known anti-apoptotic function, consisting in attenuated p53-mediated responses upon blockade of rRNA synthesis induced by chemotherapy. These findings provide the rationale for novel, specific combination strategies aimed at overcoming BCL-2 induced chemoresistance in DLBCL.

### Limitations of the study

While our findings are robust and supported by well-characterized cellular models with overexpression and knockdown approaches, PDX mouse model and comprehensive patient analyses, it is important to acknowledge possible inherent limitations, which are common in this type of study. The sample size, while sufficient for preliminary insights, may not fully represent the broader large B cell lymphoma population, thus potentially affecting the generalizability of the results. Future studies investigating the prognostic impact of nucleolar size in larger patient cohorts will be crucial to further substantiate our findings. Additionally, the inherent constraints of *in vitro* and *in vivo* models may not fully capture the complexity of lymphoma biology in the clinical setting. Finally, the precise mechanism by which BCL-2 overexpression regulates rRNA synthesis requires further elucidation.

## Resource availability

### Lead contact

Further information and requests for resources and reagents should be directed to and will be fulfilled by the lead contact, Prof. Enrico Derenzini, (enrico.derenzini@ieo.it).

### Materials availability

This study did not generate any new unique reagents and components. The shRNA oligonucleotide sequences and primer sequences for the target genes are provided in the STAR Methods section.

### Data and code availability

• The RNA-seq data used in this study are available at Array Express and are publicly available as of the date of publication. Publicly available datasets used in this study can be found in the Gene Expression Omnibus (GEO) database. Accession numbers are listed in the key resources table. Normalized T-GEP with associated outcome information are available upon request from the corresponding author.
• The code used in this study has been deposited on Github (https://github.com/veramazzara/BCL2_rRNA_DLBCL/tree/main).
• Any additional information required to reanalyze the data reported in this paper is available from the lead contact upon request.

## Acknowledgments

We thank the Genomic Unit, Computing Data, and Digital Research Platforms Unit, Imaging Unit, and Flow Cytometry Unit at IEO for their technical assistance. In particular, Simona Rodighero and Simona Ronzoni provided useful discussions and experimental support.

Funding: Banca del Piemonte, Fondazione Venesio, Piaggio research grant to C.T. and E.D., AIRC 5×1000 grant no. 21198 to S.P., and AIRC IG 20109 and 10.13039/501100003196Italian Ministry of Health to F.B.

## Author contributions

A.R. designed research studies, conducted experiments, acquired and analyzed data, and wrote the manuscript. S.M. conceptualized, designed and performed bioinformatic analyses, and critically revised the manuscript. D.S., S.Z., and E.B. conducted experiments and acquired the data. M.R.S. performed T-GEP. S.O., G.T., and P.F. conducted *in vivo* experiments. A.D. analyzed T-GEP data. C.Ceccarelli and C.A. performed immunohistochemistry. G.M. and F.M. performed T-GEP. V.T. performed immunohistochemistry. D.T. and M.P. performed nucleolar characterization with silver staining and analyzed the data. C. Corsini helped with *in vivo* experiments. A.C. helped with T-GEP experiments and analyzed the data. R.C., F.B., U.V., P.L.Z., C.T., and S.P. helped with data interpretation and critically revised the manuscript. E.D. conceptualized the research, designed research studies, analyzed data, and wrote the manuscript.

## Declaration of interests

E.D., research funding: Takeda, ADC-Therapeutics, and Incyte; speaker’s bureau: Roche, Incyte, and Abbvie; advisory board: Astra Zeneca, Lilly, Abbvie, Roche, Gilead, Takeda, and Sobi. F.B., research support: Roche and Menarini; speaker’s bureau: Pfizer. P.L.Z., consultant: MSD, Eusapharma, and Novartis; speaker’s bureau: Celltrion, Gilead, Jassen-Cilag, BMS, Servier, MSD, Astrazeneca, Tekada, Roche, Eusapharma, Kyowa Kirin, Novartis, Incyte, and BeiGene; advisory board: Secura Bio, Celltrion, Gilead, Jassen-Cilag, BMS, Servier, Sandoz, MSD, Astrazeneca, Tekada, Roche, Eusapharma, Kyowa Kirin, Novartis, ADC Terap., Incyte, and BeiGene. S.P., speaker’s bureau: Lilly, Takeda, BeiGene, Stemline, and Roche; advisory board: Lilly, Stemline, and Diatech.

## STAR★Methods

### Key resources table

**Table:** 

REAGENT or RESOURCE	SOURCE	IDENTIFIER
**Antibodies**		

Cleaved Caspase 3	Cell Signaling Technology	Cat #9664; RRID: AB_2070042
BCL-2	Cell Signaling Technology	Cat#4223; RRID: AB_1903909
c-MYC	AbCam	Cat#32072; RRID: AB_731658
MDM2 2A10	AbCam	Cat#ab16895; RRID: AB_2143534
VeriBlot for IP Detection Reagent (HRP)	AbCam	Cat#ab131366; RRID: AB_2892718
Beta-Actin	SIGMA	Cat#A5316; RRID: AB_476743
Vinculin	SIGMA	Cat#V9131; RRID: AB_477629
P53 DO-1	Santa Cruz Biotechnology	Cat#sc126; RRID: AB_628082
RPL11	Bio-Rad	#VMA00652
RPL-5	Bethyl	#A303933A
Nucleolin 4E2	GeneTex	Cat#GTX13541; RRID: AB_372550
anti-mouse HRP-conjugated	Biorad	Cat#170-6515; RRID: AB_11125142
anti-rabbit HRP-conjugated	Biorad	Cat#170-6516; RRID: AB_11125547
BCL-2 E17	AbCam	#32124
MDM2 SMP14	AbCam	#263453
BCL-2 clone 124	Dako Agilent	Cat#M0887; RRID: AB_2064429
cMYC clone EP121	Epitomics	#MON3393
Nucleolin clone 4E2	AbCam	Cat#ab13541 RRID: AB_561053

**Biological samples**		

Cohort 1: 83 patients treated with R-CHOP/CHOP-like regimens	Real-life exploratory cohort, S. Orsola-Malpighi Hospital, Bologna (Italy), from 2007 to 2012	Study *n* 12/2009/U/Tess, protocol 148/2009
Cohort 2: 46 DLBCL patients	DLCL04 study (ref. 39)	ClinicalTrials.gov, number NCT00499018https://clinicaltrials.gov/expert-search?term=NCT00499018
Patient-derived xenografts (PDX) LNH1	Laboratory of Hematology-Oncology, IEO European Institute of Oncology IRCCS, Milan, Italy	N/A

**Chemicals, peptides, and recombinant proteins**		

DOXORUBICIN	SelleckChem	#S1208
ACTINOMYCIN-D	Biovision	#1036-50
CX-5461	SelleckChem	#S2684
NUTLIN-3	Sigma-Aldrich	#N6287
IDASANUTLIN	SelleckChem	#S7205
ETOPOSIDE	Sigma-Aldrich	#E1383
VENETOCLAX (ABT-199)	SelleckChem	#GDC-0199
VINCRISTINE	Merck Life Science	#V8879
6a-methylprednisolone 21-hemisuccinate S	Merck Life Science	#M3781
ACROLEIN	Sigma-Aldrich	#110221

**Critical commercial assays**		

Cell Titer Glo Assay	Promega	#G7573
Caspase-Glo 3/7 Assay	Promega	#G8093
Quick-RNA MiniPrep kit	ZYMO RESEARCH	#R1055
LunaScript RT SuperMix kit	Biolabs	#E3010

**Deposited data**		

Raw RNA-seq data	this paper	ArrayExpress: E-MTAB-14977
Expression profiling by array	GEO	GEO: GSE10846; GSE117556

**Experimental models: Cell lines**		

SUDHL-4, SUDHL-6, OCI-LY-7, SUDHL10, OCI-LY-19, OCI-LY-18, OCI-LY-1	DSMZ	ACC 495, ACC 572, ACC 688, ACC 576, ACC 528, ACC 699, ACC 722
SUDHL-5, SUDHL2, Pfeiffer	ATCC	CRL-2958, CRL-2956, CRL-2632
TMD8, HBL-1	Dr. A. Younes, Memorial Sloan Kettering Cancer Center, NY	N/A

**Experimental models: Organisms/strains**		

Mice: NOD.Cg-PrkdcSCIDIl2rgtm1Wjl/SzJ (NSG)	Charles River Laboratories	#005557

**Oligonucleotides**		

BCL2sh1: Fw:ACCGGTGGATGACTGAGTACCTGAACGTTAAT ATTCATAGCGTTCAGGTACTCAGTCATCCATTTT Rv:CGAAAAAATGGATGACTGAGTACCTGAACGCTA TGAATATTAACGTTCAGGTACTCAGTCATCCAC	This paper	N/A
BCL2sh2: Fw:ACCGGGTGATGAAGTACATCCATTATGTTAAT ATTCATAGCATAATGGATGTACTTCATCACTTTT Rv:CGAAAAAAGTGATGAAGTACATCCATTATGCT ATGAATATTAACATAATGGATGTACTTCATCACC	This paper	N/A
MYC = Fw TTCGGGTAGTGGAAAACCAG; Rv CAGCAGCTCGAATTTCTTCC	This paper	N/A
ACTIN = Fw GAACGGTGGTGTGTCGTTC; Rv GCGTCTCGTCTCGTCTCACT	This paper	N/A
BCL-2 = Fw GAGTTCGGTGGGGTCATGT; Rv GCCGGTTCAGGTACTCAGTC	This paper	N/A
rRNA45S = Fw GCGTCTCGTCTCGTCTCACT; Rv GAACGGTGGTGTGTCGTTC	This paper	N/A
TP53 = Fw GAGCTGAATGAGGCCTTGGA; Rv CTGAGTCAGGCC CTTCTGTCTT	This paper	N/A
CDKN1A = Fw AAATCGTCCAGCGACCTTCCTCAT; Rv TCTGACTCCTTGTTCCGCTGCTAA	This paper	N/A
PUMA = Fw TTACTTCCTGCCCTGCTCTGGTTT; Rv TCTCAGGAGGTGCACGTTTCATCA	This paper	N/A
TBP = Fw CACGAACCACGGCACTGATT; Rv TTTTCTTGCTGCCAGTCTGGAC	This paper	N/A

**Recombinant DNA**		

InDOXible Tet-Activated cDNA Lentiviral Expression System	Cellecta	CUSTOM
shRNA Lentiviral Vector with Tet-Inducible U6 Promoter	Cellecta	#SVSHU6T16-L

**Software and algorithms**		

FastQC version 0.11.9	https://www.bioinformatics.babraham.ac.uk/projects/fastqc/	
BBDUk- BBMap version 38.79	https://archive.jgi.doe.gov/data-and-tools/software-tools/bbtools/bb-tools-user-guide/bbduk-guide/	
STAR version 2.7.3a	https://github.com/alexdobin/STAR	
R version 4.1.1	https://www.r-project.org/	
Github	https://github.com/veramazzara/BCL2_rRNA_DLBCL/tree/main	
ImageJ(Fiji) version 1.52	ImageJ	https://fiji.sc/
BD FacsDIVA v8.0.1.1	BDBiosciences	https://www.bdbiosciences.com/en-us/products/software/instrument-software/bd-facsdiva-software
LasX version 3.5.5	Leica Mycrosystems GmbH	https://www.leica-microsystems.com/products/microscope-software/p/leica-las-x-ls/
GraphPad Prism version 10.4.0	GraphPad Software, CA, USA	https://www.graphpad.com/features
FlowJo version 9.3	FlowJo, LLC	https://www.flowjo.com/

### Experimental model and study participant details

#### Cell lines

The human DLBCL-derived cell lines SUDHL-4, SUDHL-6, OCI-LY-7, SUDHL10 and OCI-LY-19, OCI-LY-18, OCI-LY-1 were obtained from the DSMZ-German Collection of Microorganisms and Cell Cultures, Department of Human and Animal Cell Cultures (Braunschweig, Germany); SUDHL-5, SUDHL2, Pfeiffer were obtained from ATCC (American Type Culture Collection). The DLBCL-derived cell lines, TMD8 and HBL-1, were provided by Dr. A. Younes (Memorial Sloan Kettering Cancer Center, NY).

Cell lines were cultured in RPMI 1640 medium or in Iscove Modified Dulbecco’s medium supplemented with 10–20% heat-inactivated fetal bovine serum, 1% L-glutamine, and penicillin-streptomycin in a humid environment of 5% CO2 at 37°C.

Cells were checked for mycoplasma contamination by PCR.^55^ Cell lines were authenticated by STR DNA typing (StemElite ID system) using gene amp PCR System 9700 thermal cycler (Applied biosystem) for STR amplification.

#### Mice

Experiments involving animals were approved by the Italian Ministry of Health and were performed in accordance with Italian Law (D.lgs. 26/2014 and amendments), Institutional Animal Care and Use Committee, institutional guidelines of the European Institute of Oncology, and ARRIVE guidelines. Mice were bred and housed under pathogen-free conditions in an animal facility at the IEO–IFOM Campus (Milan, Italy). *In vivo* studies were performed using 10-week-old immunodeficient NOD.Cg-PrkdcSCIDIl2rgtm1Wjl/SzJ (NSG) mice (Charles River Laboratories, Italy), with an equal distribution of male and female subjects.

#### Patients and study approval

In the present study, we analyzed two independent patient cohorts.

##### Cohort 1

83 patients from a real-life exploratory cohort treated with R-CHOP/CHOP-like regimens at S. Orsola-Malpighi Hospital, Bologna, from 2007 to 2012 (Study *n* 12/2009/U/Tess, protocol 148/2009). The patients enrolled in this study were selected based on the availability of residual tissue for AgNOR staining. However, only 56 patients from cohort 1 had tissue available for nucleolin immunohistochemistry.

##### Cohort 2

46 patients from the DLCL04 study,^39^ a prospective randomized phase 3 clinical trial investigating the role of first-line autologous stem cell transplant consolidation after chemoimmunotherapy in CD20^+^ DLBCL (ClinicalTrials.gov, number NCT00499018). All patients had available tissue for immunohistochemistry, FISH, T-GEP, and silver staining of the Nucleolar organizer regions (NOR).

Patient characteristics are summarized in Table 1. This study was approved by the Institutional Review Boards of the participating centers in accordance with the Declaration of Helsinki.

### Method details

#### BCL2 inducible system

Inducible overexpression of the coding sequence of BCL2 in the SUDHL5 (BCL2 negative) DLBCL cell line was performed using the Cellecta InDOXible Tet-Activated cDNA Lentiviral Expression System (custom Cellecta). Two lentiviral vectors, the inducible cDNA, which is under the control of the responsive promoter (TRE) and the transactivator (rtTA), were transduced separately into SUDHL-5 and OCI-LY-7 cells, allowing gene overexpression after tetracycline analog doxycycline induction. The transactivator vector contained a puromycin resistance gene (Puro), while the TRE-Empty, the TRE-BCL2 (containing the BCL2 coding sequence) and the TRE-BCL2-3′UTR (comprising both the BCL2 coding sequence and their regulatory 3′UTR region) vectors contained a green fluorescent protein (GFP) mark (Ubi-GFP).

#### BCL2 downregulation

Two ShRNAs (shRNAs) targeting BCL2 were designed and cloned into an inducible lentiviral vector (Cellecta) encoding green fluorescent protein (GFP) as a reporter gene and a Tet-On system allowing inducible shRNA expression upon doxycycline treatment (1ug/ml). These shRNA was used to specifically downregulate BCL2 expression in TMD8 and OCILY19 lymphoma cell lines. The efficiency of BCL-2 knockdown was confirmed by qPCR and Western blot analysis. The two shRNA sequences are listed below:

BCL2sh1:

Fw: ACCGGTGGATGACTGAGTACCTGAACGTTAATATTCATAGCGTTCAGGTACTCAGTCATCCATTTT.

Rv: CGAAAAAATGGATGACTGAGTACCTGAACGCTATGAATATTAACGTTCAGGTACTCAGTCATCCAC.

BCL2sh2:

Fw: ACCGGGTGATGAAGTACATCCATTATGTTAATATTCATAGCATAATGGATGTACTTCATCACTTTT.

Rv: CGAAAAAAGTGATGAAGTACATCCATTATGCTATGAATATTAACATAATGGATGTACTTCATCACC.

#### qPCR assays

Total RNA was extracted with the ZYMO RESEARCH Quick-RNA MiniPrep kit protocol. A total of 1μg of RNA was converted to cDNA using LunaScript RT SuperMix kit (Bio-Labs) following the manufacturer’s protocol. Quantitative polymerase chain reaction (qPCR) was performed using the Fast SYBR Green master mix (Applied biosystems) in a ViiA7 Real-Time PCR System (Bio-Rad). Relative RNA enrichment was calculated using the ΔΔCt method, normalized to B-actin. Specific primers were designed and listed below:

MYC = Fw TTCGGGTAGTGGAAAACCAG; Rv CAGCAGCTCGAATTTCTTCC.

ACTIN = Fw GAACGGTGGTGTGTCGTTC; Rv GCGTCTCGTCTCGTCTCACT.

BCL-2 = Fw GAGTTCGGTGGGGTCATGT; Rv GCCGGTTCAGGTACTCAGTC

rRNA45S = Fw GCGTCTCGTCTCGTCTCACT; Rv GAACGGTGGTGTGTCGTTC.

TP53 = Fw GAGCTGAATGAGGCCTTGGA; Rv CTGAGTCAGGCC CTTCTGTCTT.

CDKN1A (P21) = Fw AAATCGTCCAGCGACCTTCCTCAT; Rv TCTGACTCCTTGTTCCGCTGCTAA.

PUMA = Fw TTACTTCCTGCCCTGCTCTGGTTT; Rv TCTCAGGAGGTGCACGTTTCATCA.

TBP = Fw CACGAACCACGGCACTGATT; Rv TTTTCTTGCTGCCAGTCTGGAC.

#### Co-immunoprecipitation (Co-IP)

For immunoprecipitation, cells were lysed on ice in immunoprecipitation buffer (25 mM Tris HCl [pH 7.5], 150 mM KCl, 5 mM MgCl2, 1 mM EGTA, 1mM DTT, 10% glycerol, 0.8% Igepal/NP40, and protease inhibitors PMSF, Leupeptin and Aprotinin). The lysates were cleared by centrifugation and quantified by Bradford protein assay (Bio-Rad). 2 mg of proteins were incubated at 4°C with rotation overnight in immunoprecipitation buffer with anti-MDM2 (SMP14) or Mouse IgG Abs. ProteinA-coated agarose beads (Santa Cruz) were added to the extracts and mixed by rotation for an additional 2h at 4°C. Finally, the beads were washed four times with immunoprecipitation buffer and resuspended in protein loading buffer for western blot analysis. 5% of total lysates was used al INPUT.

#### Immunoprecipitation-qPCR (IP-qPCR)

Nucleolin immunoprecipitation was performed to suit protein-RNA interaction studies to evaluate the enrichment of BCL2 mRNA associated with nucleolin. Briefly, cells were lysed in PLB buffer (10 mM HEPES, pH 7, 100 mM KCl, 5 mM MgCl2, 0,5% NP40, 1nM DTT, RnaseOut and protease inhibitor cocktail). Following centrifugation, the supernatant was pre-cleared with Protein A Sepharose beads to minimize non-specific binding and quantified. Nucleolin was immunoprecipitated from 2mg lysate by incubating the pre-cleared lysate with 5 μg of anti-Nucleolin antibody (Cell Signaling D4C70) overnight at 4°C with gentle rotation. Immune complexes were then captured by adding Protein A Sepharose beads and incubating for 2 h at 4°C. Beads were washed sequentially with NT2 washing buffer (50 mM Tris, pH 7.4, 150 mM NaCl, 1 mM MgCl2, 0,05% NP40, 0.1nM DTT and RnaseOut) to reduce background signal.

Following IP, RNA was extracted from the bead-bound immune complexes using ZYMO RESEARCH Quick-RNA MiniPrep kit according to the manufacturer’s instructions. RNA concentration and purity were assessed using a NanoDrop spectrophotometer. The extracted RNA was reverse transcribed into cDNA using the LunaScript RT SuperMix kit (Bio-Labs) and quantitative PCR (qPCR) was performed using the Fast SYBR Green master mix (Applied biosystems) in a ViiA7 Real-Time PCR System (Bio-Rad). Relative RNA enrichment was calculated using the ΔΔCt method, normalized to input RNA and a negative IgG control.

#### RNA sequencing

Following pre-treatment with doxycycline 1 μg/ml for 96 h, SUDHL-5 cells transfected with empty vector (Empty) or BCL-2 TET-ON inducible system (BCL-2), were incubated for 6h with Doxorubicin 100nM, Act D 2.5nM, CX-5461 2500nM. Total RNA extraction was performed using ZYMO RESEARCH Quick-RNA MiniPrep kit and libraries prepared using TruSeq RNA Sample Preparation Kit (Illumina) following manufacturer instructions. Bioanalizer 2100 (Agilent Technologies) was used to qualitatively and quantitatively check prepared libraries. Final libraries, prepared in triplicate, were sequenced on an Illumina Hi-Seq 2000with paired-end 2 x 51-bp read lengths.

Quality control of the reads was performed using the FastQC software. Adapter removal and trimming was performed using BBDuk-BBMap. The reads were aligned to the reference genome (GRCh38) using STAR software. The STAR –quantMode GeneCount option was used to output a ReadSPerGene count file for each sample. The computed output was then used as the input for the DESeq2 analysis. RNA counts were normalized and used for PCA. To estimate the differential expression, the gene count matrix was analyzed using DESeq2. Upregulated and downregulated genes were selected by setting a false discovery rate lower than 5%. Pathway enrichment analysis using the KEGG database was performed using ClusterProfiler.

#### *In vivo* experiment

*In vivo* studies were performed using immunodeficient NOD.Cg-PrkdcSCIDIl2rgtm1Wjl/SzJ (NSG) mice (Charles River Laboratories, Italy). Subcutaneous tumors were grown in the flanks of mice by injecting 0.3×10^6^ primary tumor cells derived from a DLBCL patient (LNH1). Tumor growth was monitored twice a week using a digital caliper, and tumor volume was calculated according to the formula L × W2/2 (mm^3^), where W represents the width and L is the length of the tumor mass. Treatments were started approximately 10 days after tumor injection, when a palpable tumor was detected in each mouse.

The experimental treatment was performed for 2 weeks as follows: Vehicle, Act D 0.04 mg/kg, venetoclax 50 mg/kg, idasanutlin 100 mg/kg, and the combinations (Act D + venetoclax; Act D + idasanutlin; venetoclax + idasanutlin; Act D + venetoclax + idasanutlin). Treatments were administered five days/week for 14 days, and each group contained five mice. Tumor volume was measured using a caliper every 5 days.

Act D was dissolved in dimethyl sulfoxide (DMSO) and diluted with PBS. Venetoclax was prepared weekly in 10% ethanol, 30% polyethyleneglycol-400, and 60% phosal 50 propylene glycol. Idasanutlin was formulated weekly in 60% water, 30% polyethyleneglycol-400, 5% tween-80 and 5% DMSO. The mice were observed daily throughout the treatment period for signs of morbidity/mortality.

#### *In vitro* proliferation assay

Cells were seeded in 96-well plates at 25,000cell/100μL/well with either DMSO 0.1% or increasing concentrations/combinations of drugs for 24 and 48 h. Cell viability was assessed by adding Cell Titer Glo reagent (Promega) to the culture medium, according to manufacturer’s instructions.

#### Caspase 3/7 activity assay

Cells were seeded in 96-well plates at 25,000cell/100μL/well with either vehicle (DMSO 0.1%) or drugs for 12 h. Measurements of caspase activities in cells were performed using the commercially available Caspase-Glo 3/7 Assay (Promega), according to the manufacturer’s instructions.

#### Western blotting

Preparation of cellular protein lysates was performed by using the Cell Signaling lysis buffer (#9803) according to manufacturer’s extraction protocol. A total of 30 μg of protein was denatured in Laemmli buffer at 95°C for 5 min and western immunoblotting was performed using the Biorad system (TGX 4–15% gels). Transfer was performed using the Trans Blot turbo system (Bio-Rad) onto Nitrocellulose membranes. Images were acquired by using the BioRad Imaging Chemidoc MP system. The antibodies used are listed in the key resources table.

#### Cell cycle analysis

Single cell suspensions were prepared and fixed with 70% fresh Ethanol for 2 h. After fixation, cells were washed twice with 1x PBS, 1% BSA. Cells were then stained with Propidium Iodide (PI Sigma) 10 μg/ml + RNase overnight, protected from light before analysis. Data Acquisition: Flow cytometric data were acquired on a BD FACS Celesta using BD FacsDIVA Software. Analysis was performed using Flow.Jo 9.3.

#### Nucleolin immunofluorescence staining and confocal laser microscopy

500.000 SUDHL5 Empty or BCL2 cells were fixed on polylysined slides using 4% paraformaldehyde for 20 min at room temperature. To detect C23 protein, cells were treated with Triton X-100 0,5% (Sigma-Aldrich) and washed with phosphate buffered saline (PBS). After 30min of blocking in PBS +2% BSA, anti-Nucleolin 4E2 mouse antibody, 1/400 (GeneTex) and anti-BCL2 E17 rabbit antibody 1/100 (Abcam) were added, in a dark, humidified chamber (30 min; 37°C). After five washes, cells were incubated with secondary antibodies: anti-mouse Alexa Fluor 647 and anti-rabbit Alexa Fluor 488 1/400 (40 min; 37°C). After five washes, cells were counterstained with DAPI 1/5000 (30 min; 37°C) and mounted in Mowiol.

Images were obtained using an SP5 AOBS confocal microscope equipped with a 63X/1.4NA oil-immersion objective, PMT and HyD detectors and LasX 3.5.5 acquisition software (Leica Microsystems GmbH). Images were processed using Fiji/ImageJ software (v1.52, National Institutes of Health). Images represent a single confocal plane where brightness and contrast were optimized.

#### Immunohistochemistry

Immunohistochemistry (IHC) was performed on tissue microarrays (TMAs) prepared from paraffin blocks using a precision instrument as previously described. The following primary antibodies were used: anti-BCL2 (clone 124, Dako Agilent, dilution 1:100), anti-cMYC (clone EP121, Epitomics, dilution 1:100), TP53 (clone DO-7, Dako Agilent, dilution 1:150) and anti-Nucleolin (clone 4E2, Abcam, dilution 1:6000). For BCL2, cMYC and TP53 antigen retrieval was carried on PT-links at 92°C for 5 min in EnVision Flex Target Retrieval Solution High pH (Dako Agilent, Glostrup, Denmark). IHC tests were performed on AutoStainer Plus platforms, using the LSAB+REAL Detection System (Dako Agilent). The IHC preparations were counterstained with Gill’s hematoxylin and mounted in Kaiser’s glycerin. The cut-off values of 40%, 50% and 50% positive neoplastic cells were applied for MYC, BCL2 and TP53 respectively.^56^ Nucleolin antigen retrival was carried out in Tris-HCL Buffer pH 8,5, at 95°C for 40 min and IHC test in Benchmark Ultra immunostainer using OptiView DAB detection kit (brown color) (Ventana Diagnostic Systems, Tucson, AZ, USA). Sections were counterstained using Hematoxylin and Bluing reagent following Ventana indications. Three different nucleolin expression patterns were identified: pattern 1, in which Nucleolin appeared to be exclusively located in the nucleolus; pattern 2, in which the staining reaction, other than in the nucleolus, occurred in the nucleoplasm and sometimes homogeneously stains all the nucleus; pattern 3, in which the nuclei of all the cells were uniformly stained, thus completely masking the visualization of the nucleolus.

#### Nucleolar organizer regions silver staining and image cytometry

After silver staining the NORs are identified as black dots exclusively localized throughout the nucleolar area. NOR silver staining was performed according to the guidelines of the “International Committee on AgNOR quantitation”.^57^ Briefly, slides were moved from water to heat-resistant plastic Coplin jars, fully immersed in 10mM sodium citrate buffer (pH 6.0) and autoclaved at 120°C for 20 min. After cooling to room temperature in the sodium citrate buffer, slides were stained with silver for 13 min at 37°C in the dark using a solution of one volume 2% gelatine in 1% aqueous formic acid and two volumes of 50% silver nitrate. Sections were finally dehydrated and mounted in Canada balsam without any counterstaining.

Morphometric analysis of AgNORs was carried out using the Image-Pro Plus program (Media Cybernetics, Silver Spring, Maryland, USA). The main stages of image processing were as follows: a microscope field was selected by the operator using a ×40 objective lens. The selected image was then captured and stored in the digital memory, and displayed on the color monitor. Here, the operator interactively defined the gray threshold, which permitted automatic quantification of the black dots corresponding to the silver-stained nucleoli. The morphometric analysis was then performed on a cell-by-cell basis by converging the window over the corresponding nucleus. For each case, the AgNOR area of at least 200 nuclei was measured and the mean (SD) AgNOR area was calculated.

#### FISH analysis

MYC, BCL2 and BCL6 translocations were tested by FISH. FISH studies were conducted on paraffin sections using the following probes: (Abbott, Abbott Park, IL, USA) Vysis LSI MYC dual color break-apart, Vysis LSI BCL2 dual color break-apart, Vysis LSI BCL6 dual color break-apart. Slides were deparaffinized, co-denatured with probe, hybridized overnight, washed and then mounted with DAPI/Antifade. For each probe, 200 interphase nuclei were analyzed to detect re-arrangements and numerical abnormalities. Cut-off values were established for each probe by assessing 10 normal controls (reactive lymph nodes) and choosing values three standard deviations above the mean.

#### Targeted gene expression profiling

Digital quantification of gene expression was performed using the NanoString platform. Total RNA was extracted from three 15μm sections of each FFPE sample using the RecoverAll Total Nucleic Acid Isolation Kit for FFPE (Invitrogen). The yield of the extracted RNA was assessed using a NanoDrop ND-1000 Spectrophotometer (NanoDrop Technologies). RNA quality was scored according to the DV200 value (% of RNA fragments >200 nucleotides) using the Agilent RNA 6000 Nano Chip Kit (BioAnalyzer, Agilent Technologies). Only samples with a DV200 value > 20% passed the QC step.

Two custom panels were profiled to determine the Cell-of-Origin molecular subtype and to quantify the expression of genes belonging to the BCL-2 family. The COO/MYC-BCL2 panel contained 22 genes: 20 genes for COO subtyping plus MYC and BCL-2.^58^ To determine the COO molecular subtype, expression data were analyzed at NanoString Technologies, Inc. using a modified RUO version of the NanoString Lymphoma Subtyping Test (LST) algorithm.^13^^,^^59^^,^^60^

The LST algorithm measures the geometric mean of 5 housekeeping genes (HK geomean) to ensure RNA quality based on a pre-defined clinical QC threshold of 128 (indicated as Pass). An HK geomean value below 64 is deemed insufficient RNA quality to provide a subtyping result (listed as fail). A value between 64 and 128 is “borderline” quality since it meets previously published thresholds for RNA quality within clinical research studies but it does not meet Nanostring clinical QC threshold of 128 for individual patients.

Each sample meeting the QC threshold was assigned to one molecular subtype: Activated-B-Cell (ABC), GC-B-Cell (GCB) or Unclassified within an equivocal zone.

The quality control and normalization of NanoString nCounter were performed using R package NanoStringNorm. The raw NanoString counts for each gene were subjected to a technical normalization considering positive and negative probes. A normalization factor was calculated by obtaining the geometric mean of the positive controls used for each sample and applied to the raw counts of the nCounter output data to eliminate variability unrelated to the samples. The resulting data were normalized again with the geometric mean of the housekeeping genes. Normalized data were log2-transformed for further analyses.

#### RNA-seq data analysis

Gene expression data were derived using an automated pipeline based on anaconda environment.^61^ The analysis steps are listed in sequence.

##### Quality control

Quality check is performed using FastQC v0.11.9 and it is run for every sequence file independently

fastqc --o output_folder read1.fastq.gz read2.fast.gz.

##### Quality trimming and adapter removal

The sequence files are processed with bbduk v38.79 to remove parts of the readouts that match adapter sequences and to remove poor quality base pair readouts at the end of the reads.

bbduk.sh in1 = read1.fastq.gz in2 = read2.fastq.gz out1 = read1_trim.fastq.gz out2 = read2_trim.fastq.gz qtrim = r trimq = 20 minlen = 25.

bbduk.sh in1 = read1_trim.fastq.gz in2 = read2_trim.fastq.gz out1 = read1_cleaning.fastq.gz out2 = read2_cleaning.fastq.gz ref = adapters.fa ktrim = r k = 23 mink = 11 hdist = 1 tpe tbo.

##### Generate genome indexes

The GRCh38 human genome assembly is used as the genome target and the transcriptome target is defined by the GENCODE release 34 (basic gene annotation). Genome indexing is performed using the following command.

STAR --runThreadN 8 --runMode genomeGenerate –genomeDir genome_folder --genomeFastaFiles GRCh38.primary_assembly.genome.fa --sjdbGTFfile gencode.v34.primary_assembly.annotation.gtf --sjdbOverhang 50.

##### Mapping and quantification

Reads mapping was performed using STAR v2.7.3a. The STAR -quantMode GeneCount option was used for gene quantification, with the resulting strand counts chosen depending upon library preparation (unstranded).

STAR –runThreadN 8 --runMode genomeGenerate --genomeDir indices_folder –genomeFastaFiles GRCh38.primary_assembly.genome.fa --sjdbGTFfile gencode.v34.primary_assembly.annotation.gtf --sjdbOverhang 50.

#### Differential expression analysis

STAR read counts were used as input into DESeq2.^62^ Genes with raw counts greater than 20 were kept for subsequent differential expression gene (DEG) analyses. Mitochondrial genes were removed from the downstream analysis. DEG analysis was performed with drug inhibiting ribosome biogenesis (doxorubicin, Act D, CX-5461) and BCL-2 status (presence or absence) as the covariates of interest and DEG were called based upon a false discovery rate less than 0.05 (Benjamini-Hochberg correction). Raw counts were normalized using DESeq2’s function regularized transformation (rlog), and normalized counts were used to perform and visualize PCA results.

#### Kyoto Encyclopedia of Gene and Genome pathway analysis of differential expressed genes

To better understand the gene functions, the KEGG enrichment analysis of the DEGs was performed using clusterProfiler package.^63^^,^^64^ We first converted the gene ID from the Official Symbol of the differential genes obtained by org.Hs.e.g.,.Db package using clusterProfiler’s function ‘bitr’ followed by a KEGG analysis. Adjusted *p*-value (Benjamini-Hochberg method) has been used for terms ranking and selection (padj<0.05).

#### p53 targets: Identification and fold changes evaluation

In order to quantify the p53-mediated response after treatment with RBi in SUDHL-5 cells, we merged the six different DEGs lists in a consensus list and queried TRANSFAC database containing a list of direct p53 targets. Starting from these direct p53 targets (*n* = 256), we applied a two-step filter.

(1) only those genes with *log*_*2*_ *|fc| ≥ 1* in DMSO were considered
(2) only those genes with *|Δfc| = |fc*_*Empty*_ *- fc*_*BCL-2*_ *|≥0.58* (where *fc* is expressed in log_2_) in each treatment arm were selected

The two-step filter allowed to highlight only the significantly regulated p53 targets. At this point, levels of fold change (log_2_) of significantly regulated p53 targets identified in SUDHL-5 cells treated with RBi in presence versus absence of BCL-2 were visualized using radar charts.

### Quantification and statistical analysis

Quantitative data were collected from at least three independent experiments and are expressed as mean ± SD. Student’s t test was used to assessed significant differences between groups. The statistical details are described in the figures and in their corresponding legends for individual experiments.

For survival analysis, we used the Kaplan-Meier method to estimate OS and PFS. A log rank test assessed significant differences between curves, and the threshold to stratify patients into low- and high-risk groups was determined using the median value. Multivariate and univariate analyses were performed using the Cox proportional hazards regression model. Associations and differences in patient characteristics were analyzed using the χ^2^ and Fisher’s exact test. *p* ≤ 0.05 was considered statistically significant.

### Additional resources

The 46 patients from the DLCL04 study,^39^ were enrolled in the ClinicalTrials.gov, number NCT00499018 (https://clinicaltrials.gov/expert-search?term=NCT00499018).

## Supplemental information

Document S1. Figures S1–S9 and Table S1

Data S1. RNA-seq differential expression analysis The multiple tables provide the result of the differential expression analysis for all pairwise comparisons. Only genes with *p*adj <0.05 were reported in the lists

Data S2. Apoptotic enrichment in the empty vector SUDHL-5 cells The table provides the list of 37 genes exclusively regulated in empty vector-transduced SUDHL-5 cells related to the apoptotic pathway. The p53 direct targets are also highlighted in the table

Data S3. List of genes and target sequences for the BCL-2 T-GEP panel and COO T-GEP panel The table shows the list of genes and the associated target sequences for the two custom nanostring panels. The first one is a BCL2-family panel including all BCL-2 family members (23 genes) and the second one is 22-panel genes specific for COO subtyping and measurements of MYC and BCL-2 mRNA levels
