# Supplementary material for: Downregulation of rRNA synthesis by BCL-2 induces chemoresistance in diffuse large B cell lymphoma
Source: iScience. 2025 Apr 2;28(5):112333. doi: 10.1016/j.isci.2025.112333 (PMC12020883; doi:10.1016/j.isci.2025.112333)
Supplement: Document S1. Figures S1–S9 and Table S1 [file mmc1.pdf]

iScience, Volume *28*

Supplemental information

Downregulation of rRNA synthesis

by BCL-2 induces chemoresistance

in diffuse large B cell lymphoma

Alessandra Rossi, Saveria Mazzara, Dorotea Salemi, Simone Zanetti, Maria Rosaria Sapienza, Stefania Orecchioni, Giovanna Talarico, Paolo Falvo, Alessandro Davini, Claudio Ceccarelli, Giovanna Motta, Federica Melle, Valentina Tabanelli, Claudio Agostinelli, Davide Trerè, Marianna Penzo, Chiara Corsini, Elena Baiardi, Angelica Calleri, Umberto Vitolo, Francesco Bertolini, Pier Luigi Zinzani, Roberto Chiarle, Corrado Tarella, Stefano Pileri, and Enrico Derenzini

Figure S1

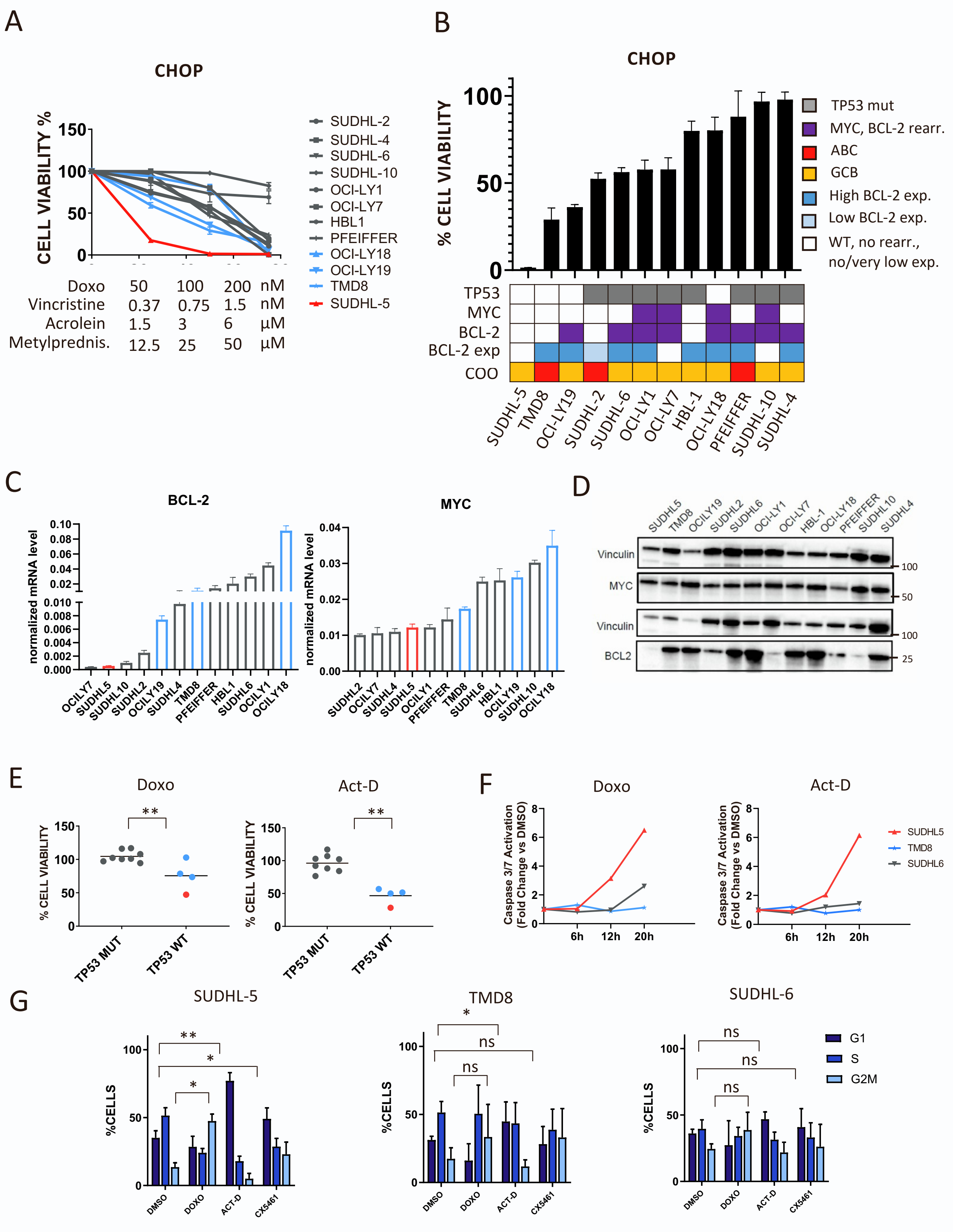
A
CHOP
CELL VIABILITY %
Doxo 50 100 200 nM
Vincristine 0.37 0.75 1.5 nM
Acrolein 1.5 3 6 µM
Metylprednis. 12.5 25 50 µM
SUDHL-2
SUDHL-4
SUDHL-6
SUDHL-10
OCI-LY1
OCI-LY7
HBL1
PFEIFFER
OCI-LY18
OCI-LY19
TMD8
SUDHL-5
B
CHOP
% CELL VIABILITY
TP53 mut
MYC, BCL-2 rearr.
ABC
GCB
High BCL-2 exp.
Low BCL-2 exp.
WT, no rearr., no/very low exp.
TP53
MYC
BCL-2
BCL-2 exp
COO
SUDHL-5 TMD8 OCI-LY19 SUDHL-2 SUDHL-6 OCI-LY1 OCI-LY7 HBL-1 OCI-LY18 PFEIFFER SUDHL-10 SUDHL-4
C
BCL-2
normalized mRNA level
OCILY7 SUDHL5 SUDHL10 SUDHL2 OCILY19 SUDHL4 TMD8 PFEIFFER HBL1 SUDHL6 OCILY1 OCILY18
MYC
SUDHL2 OCILY7 SUDHL4 SUDHL5 OCILY1 PFEIFFER TMD8 SUDHL6 HBL1 OCILY19 SUDHL10 OCILY18
D
SUDHL5 TMD8 OCI-LY19 SUDHL2 SUDHL6 OCI-LY1 OCI-LY7 HBL-1 OCI-LY18 PFEIFFER SUDHL10 SUDHL4
Vinculin
MYC
Vinculin
BCL2
100
50
100
25
E
Doxo
Act-D
% CELL VIABILITY
TP53 MUT
TP53 WT
F
Doxo
Act-D
Caspase 3/7 Activation (Fold Change vs DMSO)
6h 12h 20h
SUDHL5
TMD8
SUDHL6
G
SUDHL-5
TMD8
SUDHL-6
%CELLS
DMSO DOXO ACT-D CX5461
G1
S
G2M
ns

Figure S1: BCL-2 overexpression promotes resistance to inhibition of ribosome biogenesis in Diffuse Large B-cell Lymphoma cell lines

A) Cell Titer Glo assay (CTG) showing the effect on cell viability of increasing doses of CHOP in 12 DLBCL cell lines. *TP53* mutated cell lines are indicated in gray, *TP53* wt/BCL-2 positive cell lines in blue and the *TP53* wt/BCL-2 negative cell line SUDHL-5 is indicated in red. Cells were incubated for 24h with the indicated treatments. Error bars represent the standard deviation (SD) of triplicate experiments (n=3).

B) Bar graph showing the antiproliferative effects of CHOP chemotherapy on 12 DLBCL cell lines, as measured by CTG assay. Cells were incubated for 24h with vincristine (0.75nM), doxorubicin (100nM), acrolein (3µM) and methylprednisolone (25µM). Error bars represent SD of triplicate experiments (n=3). The heat map shown below indicates the *TP53, MYC* and *BCL-2* status and the cell of origin (COO).

C) qPCR analysis of *BCL2* and *MYC* mRNA expression in 12 DLBCL cell lines. *TP53* mutated cell lines are indicated in gray, *TP53* wt/BCL-2 positive cell lines in blue, the *TP53* wt/BCL-2 negative cell line SUDHL-5 in red. Error bars represent SD of triplicate experiments (n=3).

D) Representative immunoblots showing the baseline expression levels of MYC and BCL-2 proteins in the DLBCL cell line panel.

E) Scatter plot representing cell viability in 12 DLBCL cell lines treated with doxorubicin (100nM) and actinomycin D (2.5nM) for 24h. Each dot represents the mean of triplicate experiments (n=3). Student's t-test: **$p<0.01$.

F) Graph showing fold change over time in caspase 3/7 activity in SUDHL-5, TMD8 and SUDHL-6 cells incubated with doxorubicin (100nM) and actinomycin D (2.5nM) for 6, 12 and 20 hours.

G) Bar graph showing the effects of RiBi inhibitors (doxorubicin 100nM, actinomycin D 2.5nM, CX-5461 2500nM) on cell cycle phases in SUDHL-5, TMD8 and SUDHL-6 cells treated for 24h. Error bars represent SD of triplicate experiments (n=3). Student's t-test: *$p<0.05$, **$p<0.01$.

Figure S2

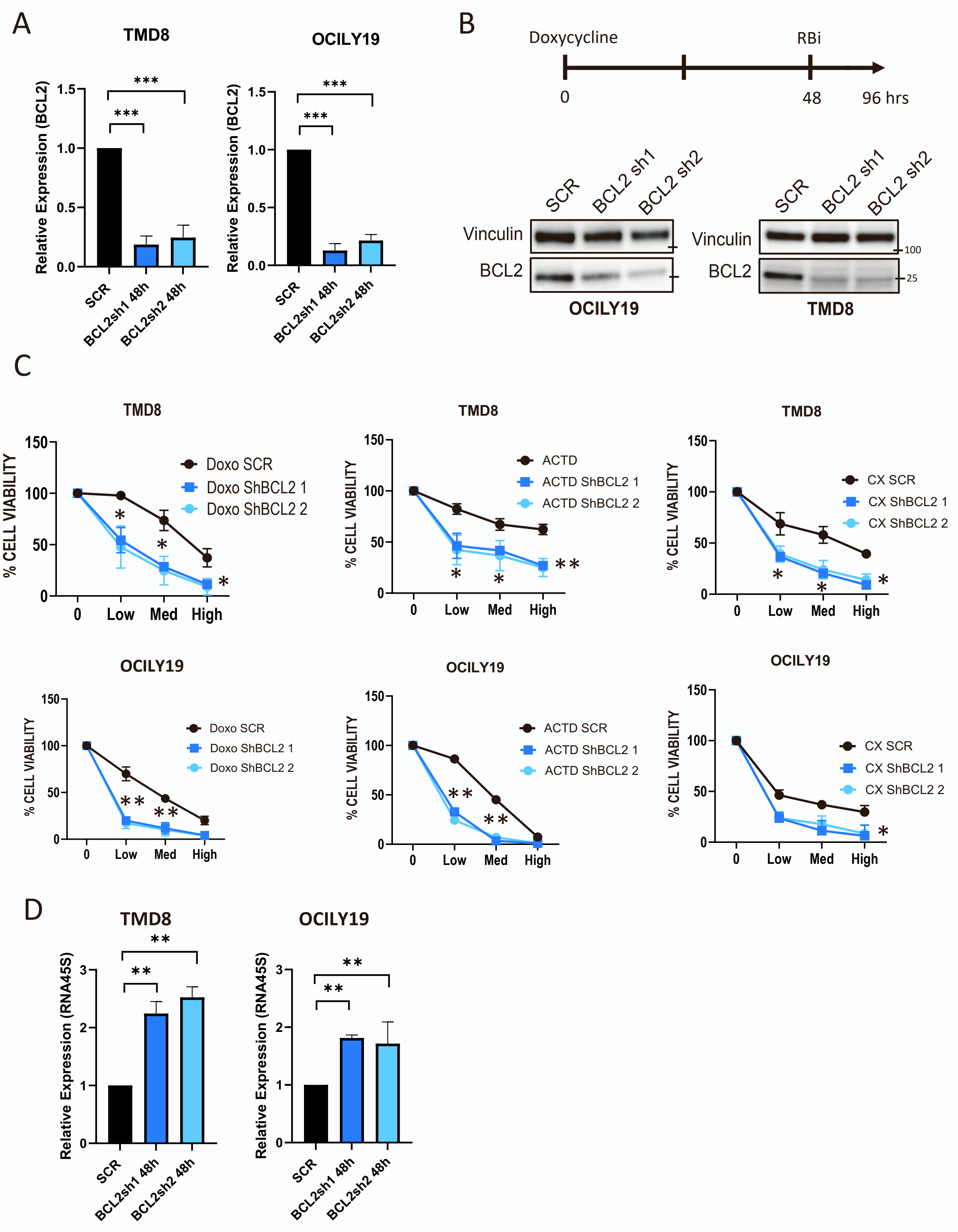
A
TMD8
OCILY19
Relative Expression (BCL2)
SCR
BCL2sh1 48h
BCL2sh2 48h
***
B
Doxycycline
RBi
0
48
96 hrs
SCR
BCL2 sh1
BCL2 sh2
Vinculin
BCL2
100
25
C
% CELL VIABILITY
Doxo SCR
Doxo ShBCL2 1
Doxo ShBCL2 2
ACTD
ACTD SCR
ACTD ShBCL2 1
ACTD ShBCL2 2
CX SCR
CX ShBCL2 1
CX ShBCL2 2
0
Low
Med
High
D
Relative Expression (RNA45S)
**

Figure S2: BCL-2 downregulation increases sensitivity to ribosome biogenesis inhibition in Diffuse Large B-cell Lymphoma cell lines

A) qPCR analysis of *BCL-2* mRNA levels in TMD8 and OCILY19 cells transduced with the scramble vector (SCR) or two different doxycycline-inducible short hairpin RNAs (shRNAs) targeting BCL2, followed by doxycycline treatment for 48h. Error bars represent SD of triplicate experiments (n=3). Student's t-test: ***p<0.005.

B) Schematic representation of the BCL-2 silencing experiments. Western blot displays BCL-2 protein expression after 48h incubation with 1µg/ml doxycycline in TMD8 and OCILY19 cells transduced with the SCR or two different doxycycline inducible shRNAs against BCL2.

C) CTG assay showing the cytotoxic effects at 48h of three doses of RiBi (doxorubicin 25, 50, 100nM, actinomycin D 0.65, 1.25, 2.5nM, CX-5461 625, 1250, 2500nM), in TMD8 and OCILY19 cells in the presence or absence of BCL-2. Error bars represent SD of triplicate experiments (n=3). Student's t-test: *p<0.05, **p<0.01, ***p<0.005.

D) qPCR analysis of 45S rRNA levels in TMD8 and OCILY19 cells transduced with the SCR or two different doxycycline inducible shRNAs targeting BCL2 followed by doxycycline treatment for 48h. Error bars represent SD of triplicate experiments (n=3). Student's t-test: ***p<0.005.

Figure S3

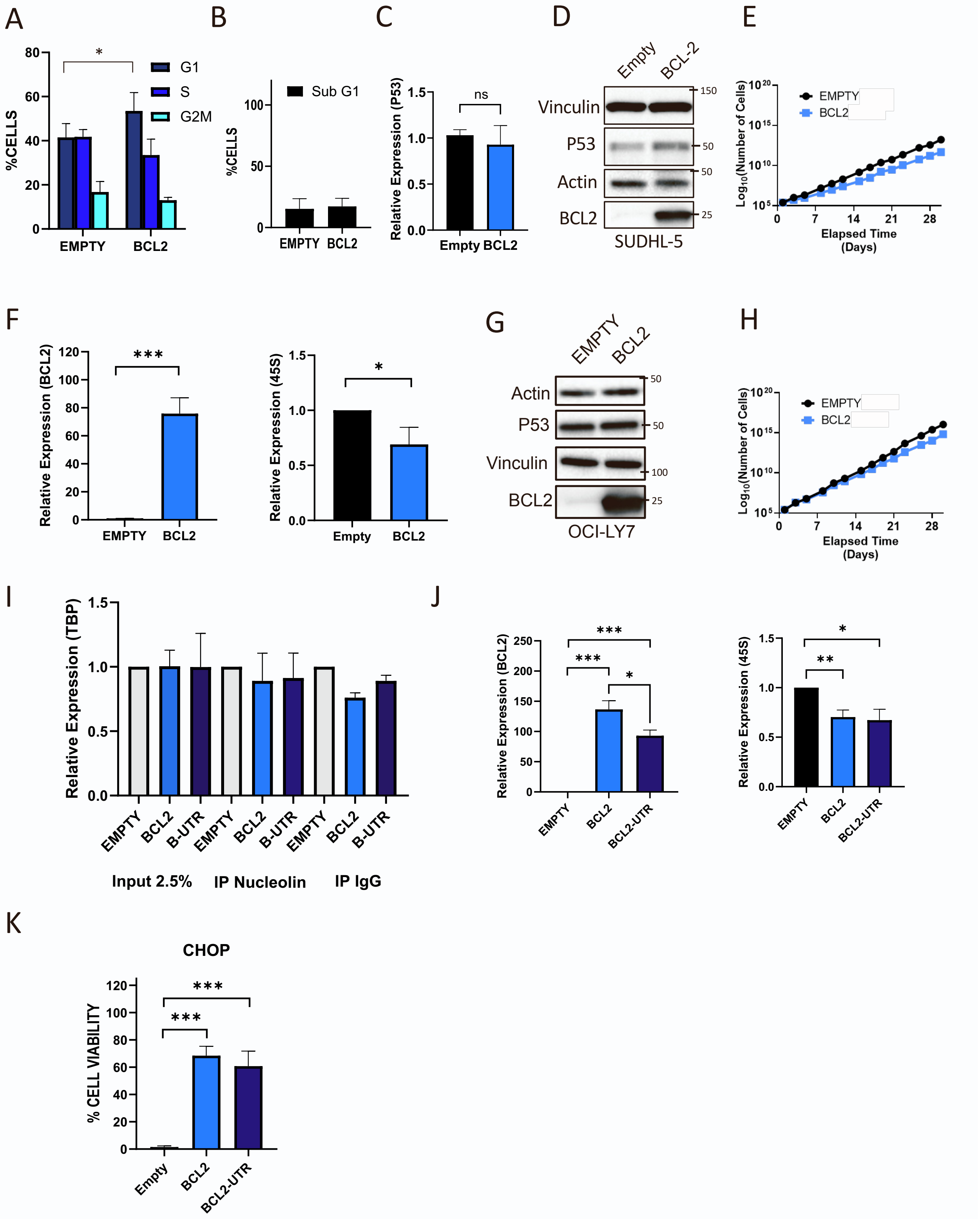
A
B
C
D
E
F
G
H
I
J
K
%CELLS
G1
S
G2M
EMPTY
BCL2
Sub G1
Relative Expression (P53)
ns
Empty
Vinculin
P53
Actin
SUDHL-5
BCL-2
Log10(Number of Cells)
Elapsed Time
(Days)
Relative Expression (BCL2)
Relative Expression (45S)
OCI-LY7
Relative Expression (TBP)
B-UTR
Input 2.5%
IP Nucleolin
IP IgG
BCL2-UTR
CHOP
% CELL VIABILITY

Figure S3: Enforced BCL-2 expression is associated with a reduced rRNA synthesis rate in DLBCL cell lines

A) Barr graph showing the effect of BCL-2 overexpression on cell cycle phases in short term culture. SUDHL-5 cells transfected with empty vector (Empty) or TET-ON BCL-2 inducible system (BCL-2) were incubated with doxycycline 1µg/ml for 24h and cell cycle phases were assessed by flow cytometry (propidium iodide staining). Error bars represent standard deviation (SD) of triplicate experiments (n=3). Student's t-test: *p<0.05.

B) Bar graph showing the effect of BCL-2 overexpression on the SubG1 fraction of the cell cycle. SUDHL-5 cells were transfected and treated as in (A), and cell cycle phases were assessed by flow cytometry (propidium iodide staining). Error bars represent SD of triplicate experiments (n=3). Differences between groups were calculated with the Student's t-test.

C) qPCR analysis of *TP53* mRNA expression in SUDHL-5 cells in the presence or absence of BCL-2. Error bars represent SD of triplicate experiments (n=3). Differences between groups were calculated with the Student's t-test.

D) Representative immunoblots showing increased p53 protein abundance in SUDHL-5 cells in the presence or absence of BCL-2.

E) Graph showing cell counts (trypan blue staining) of SUDHL-5 cells transfected with empty vector (Empty) or TET-ON BCL-2 inducible system (BCL-2), cultured with doxycycline 1µg/ml for 30 days.

F) OCI-LY7 cells (*TP53* mut/BCL-2 negative) transfected with empty vector (Empty) or TET-ON BCL-2 inducible system (BCL-2) were incubated with doxycycline 1µg/ml for 96h. *BCL-2* mRNA and *45S* rRNA levels were evaluated by qPCR analysis. Error bars represent SD of triplicate experiments (n=3). Student's t-test: *p<0.05, **p<0.01.

G) Representative western blots showing BCL-2 protein expression and p53 level in OCI-LY7 transfected with empty vector (Empty) or TET-ON BCL-2 inducible system (BCL-2), incubated with doxycycline 1µg/ml for 96h.

H) Graph showing cell counts (trypan blue staining) of OCI-LY7 cells transfected with empty vector (Empty) or TET-ON BCL-2 inducible system (BCL-2), cultured with doxycycline 1µg/ml for 30 days.

I) Immunoprecipitation-qPCR (IP-qPCR) analysis showing the nonspecific interaction between nucleolin and TBP mRNA in SUDHL-5 cells. TBP was used as a negative control. The experiment was conducted in the presence or absence of BCL-2 mRNA from the BCL-2 coding sequence (BCL2) or the BCL2 3' untranslated region (B-UTR) constructs. Cells were treated with doxycycline for 96h prior to nucleolin immunoprecipitation. qPCR analysis quantified the TBP mRNA levels in the initial sample (INPUT) and in the nucleolin-TBP mRNA complexes immunoprecipitated with a nucleolin-specific antibody (IP nucleolin). Error bars represent SD of triplicate experiments (n=3).

J) SUDHL5 cells transduced with an empty vector, BCL2 or a BCL-2 3’UTR TET-ON inducible system were incubated with 1µg/ml doxycycline for 96h. *BCL-2* mRNA and *45S* rRNA levels were assessed by qPCR analysis. Error bars represent SD of five independent experiments (n=5). Student's t-test: *p<0.05, **p<0.01.

K) CTG assay showing the cytotoxic effects of CHOP (vincristine 0.75nM, doxorubicin 100nM, acrolein 3µM, methylprednisolone 25µM) in SUDHL-5 cells in the presence or absence of BCL-2 or BCL2 3’UTR. Cells were preincubated with 1µg/ml doxycycline for 96h, followed by 24h CHOP treatment. Error bars represent SD of triplicate experiments (n=3). Student's t-test: ***p<0.005.

Figure S4

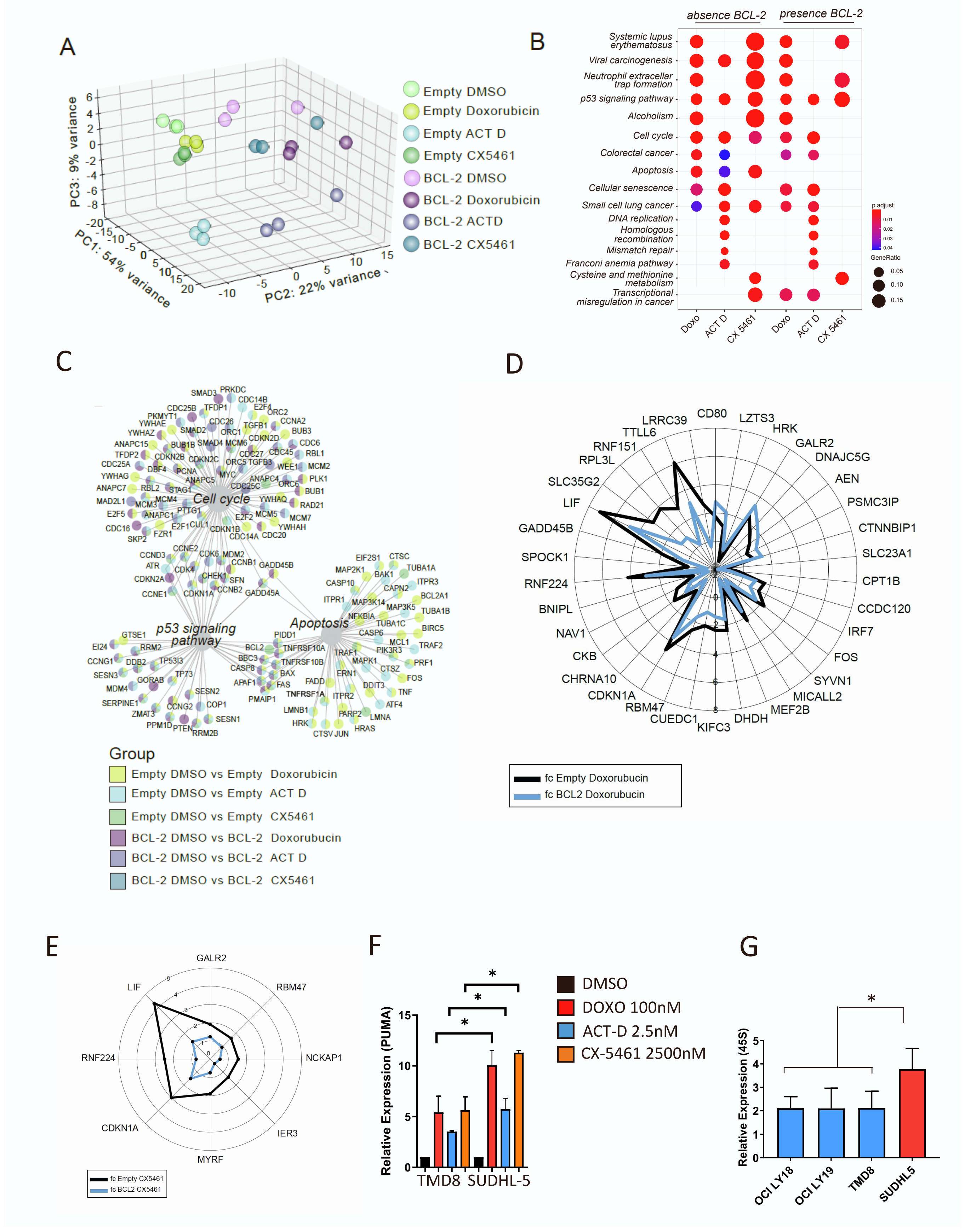
A
PC3: 9% variance
PC1: 54% variance
PC2: 22% variance
Empty DMSO
Empty Doxorubicin
Empty ACT D
Empty CX5461
BCL-2 DMSO
BCL-2 Doxorubicin
BCL-2 ACTD
BCL-2 CX5461
B
absence BCL-2
presence BCL-2
Systemic lupus erythematosus
Viral carcinogenesis
Neutrophil extracellular trap formation
p53 signaling pathway
Alcoholism
Cell cycle
Colorectal cancer
Apoptosis
Cellular senescence
Small cell lung cancer
DNA replication
Homologous recombination
Mismatch repair
Franconi anemia pathway
Cysteine and methionine metabolism
Transcriptional misregulation in cancer
Doxo
ACT D
CX 5461
p.adjust
GeneRatio
0.05
0.10
0.15
C
Cell cycle
p53 signaling pathway
Apoptosis
Group
Empty DMSO vs Empty Doxorubicin
Empty DMSO vs Empty ACT D
Empty DMSO vs Empty CX5461
BCL-2 DMSO vs BCL-2 Doxorubucin
BCL-2 DMSO vs BCL-2 ACT D
BCL-2 DMSO vs BCL-2 CX5461
D
CD80
LZTS3
HRK
GALR2
DNAJC5G
AEN
PSMC3IP
CTNNBIP1
SLC23A1
CPT1B
CCDC120
IRF7
FOS
SYVN1
MICALL2
MEF2B
DHDH
KIFC3
CUEDC1
RBM47
CDKN1A
CHRNA10
CKB
NAV1
BNIPL
RNF224
SPOCK1
GADD45B
LIF
SLC35G2
RPL3L
RNF151
TTLL6
LRRC39
fc Empty Doxorubucin
fc BCL2 Doxorubucin
E
GALR2
RBM47
NCKAP1
IER3
MYRF
CDKN1A
RNF224
LIF
fc Empty CX5461
fc BCL2 CX5461
F
Relative Expression (PUMA)
TMD8
SUDHL-5
DMSO
DOXO 100nM
ACT-D 2.5nM
CX-5461 2500nM
G
Relative Expression (45S)
OCI LY18
OCI LY19
TMD8
SUDHL5

Figure S4: BCL-2 overexpression attenuates p53 stabilization and activation following RiBi inhibitors treatment

A) Principal component analysis (PCA) of RNAseq data obtained in the experiment shown in Figure 3C.

B) Functional enrichment profiles by KEGG showing pathways regulated by RiBi inhibitors in the presence or absence of BCL-2. The dot color from red to blue represents the significance of enrichment.

C) The plot depicts the linkages of genes and KEGG pathways as a network. Cell cycle, p53 signaling and apoptosis are shown as grey nodes with edges connecting genes to corresponding pathways.

D) Radar chart displaying levels of fold changes (log2) of significantly regulated p53 targets identified in SUDHL-5 cells treated with doxorubucin 100nM for 6h in presence (blue) versus absence of BCL-2 (black). Black numbering indicates fold change (log2) levels.

E) Radar chart displaying levels of fold changes (log2) of significantly regulated p53 targets identified in SUDHL-5 cells treated with CX-5461 2500nM for 24h in presence (blue) versus absence of BCL-2 (black). Black numbering indicates fold change (log2) levels.

F) qPCR analysis showing the expression of the p53 target gene *PUMA* in TMD8 and SUDHL5 cell lines treated for 24h with doxorubicin 100nM, actinomycin D 2.5nM and CX-5461 2500nM. Error bars represent standard deviation (SD) of triplicate experiments (n=3). Student's t-test: *$p<0.05$.

G) qPCR analysis showing baseline expression levels of 45S rRNA in *TP53* wt / BCL-2 positive cell lines (blue) vs SUDHL-5 cells (red). Error bars represent SD of triplicate experiments (n=3). Student's t-test: *$p<0.05$.

Figure S5

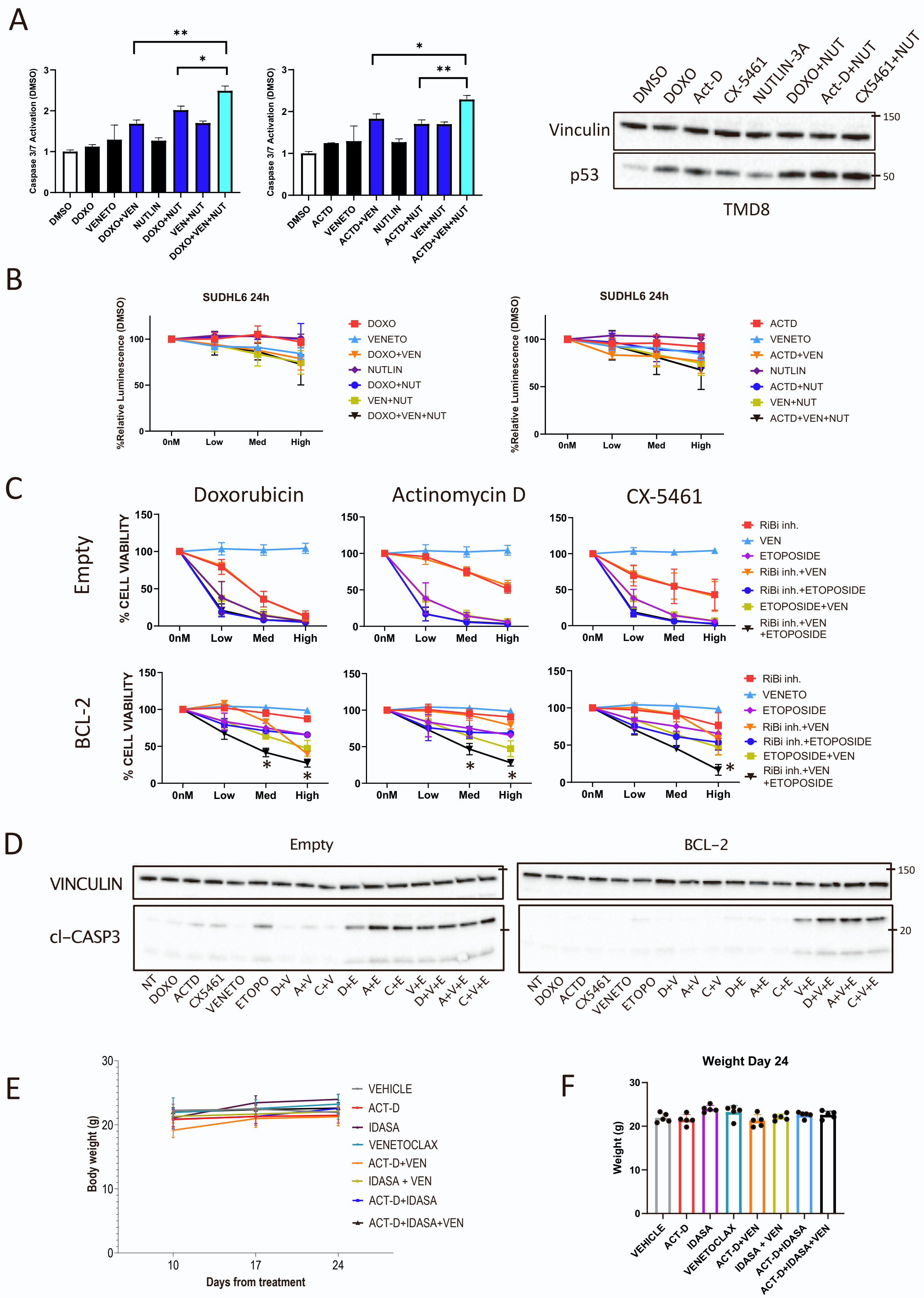

A
Caspase 3/7 Activation (DMSO)
DMSO
DOXO
VENETO
DOXO+VEN
NUTLIN
DOXO+NUT
VEN+NUT
DOXO+VEN+NUT
ACTD
ACTD+VEN
ACTD+NUT
ACTD+VEN+NUT
Vinculin
p53
Act-D
CX-5461
NUTLIN-3A
Act-D+NUT
CX5461+NUT
150
50
TMD8
B
SUDHL6 24h
%Relative Luminescence (DMSO)
0nM
Low
Med
High
DOXO+NUT
C
Doxorubicin
Actinomycin D
CX-5461
Empty
BCL-2
% CELL VIABILITY
RiBi inh.
VEN
ETOPOSIDE
RiBi inh.+VEN
RiBi inh.+ETOPOSIDE
ETOPOSIDE+VEN
RiBi inh.+VEN
+ETOPOSIDE
D
Empty
BCL–2
VINCULIN
cl–CASP3
NT
ETOPO
D+V
A+V
C+V
D+E
A+E
C+E
V+E
D+V+E
A+V+E
C+V+E
CX5461
150
20
E
Body weight (g)
Days from treatment
VEHICLE
ACT-D
IDASA
VENETOCLAX
ACT-D+VEN
IDASA + VEN
ACT-D+IDASA
ACT-D+IDASA+VEN
F
Weight Day 24
Weight (g)

Figure S5: MDM2 inhibitors in combination with venetoclax overcome BCL-2-mediated resistance to RiBi inhibitors *in vitro* and *in vivo*

A) Bar graph showing levels of caspase 3/7 activation following treatment with doxorubicin 100nM (left) or actinomycin D 2.5nM (right) as single agents or in combination with venetoclax 500nM and/or nutlin-3A 2500nM in TMD8 cells (as measured by Caspase Glo assay after 12h of incubation). Student's t-test: *$p<0.05$, **$p<0.01$. On the right, representative western blot assay showing enhanced p53 induction following treatment with RiBi inhibitors in combination with Nutlin-3A in TMD8 cells after 24h of treatment.

B) Cell Titer Glo assay (CTG) showing cell viability of the *TP53* mutated SUDHL-6 cell line treated for 24h with RiBi inhibitors (doxorubicin 25, 50, 100nM, actinomycin D 0.65, 1.25, 2.5nM), venetoclax (125, 250, 500nM) and with the MDM2i nutlin-3A (1250, 2500, 5000nM) as single agents or in combination. Error bars represent the standard deviation (SD) of triplicate experiments (n=3).

C) CTG assay showing cell viability of SUDHL-5 cells treated with RiBi inhibitors (doxorubicin 25, 50, 100nM, actinomycin D 0.65, 1.25, 2.5nM, CX-5461 625, 1250, 2500nM), venetoclax (125, 250, 500nM), etoposide (750, 1250, 2500nM) as single agents or in different combinations, in the absence or presence of BCL2. SUDHL5 cells transfected with empty vector (Empty) or BCL-2 TET-ON system (BCL-2) were treated with doxycycline 1µg/ml for 96h and then incubated with the indicated treatments for 24h. Error bars represent SD of triplicate experiments (n=3). Student's t-test: *$p<0.05$.

D) Representative western blot assays showing levels of caspase 3 activation in SUDHL-5 cells Empty or BCL2 incubated 24h with the indicated treatments: doxorubicin 100nM, actinomycin D 2.5nM, CX-5461 2500nM, venetoclax 500nM, etoposide 750nM and the combinations. D (doxorubicin), A (actinomycin D), C (CX-5461), V (venetoclax), E (etoposide).

E) *In vivo* combination experiment in a subcutaneous TP53 wt/BCL-2 positive DLBCL PDX model (LNH1). NSG mice were treated with vehicle, 0.04mg/kg actinomycin D, 50mg/kg venetoclax and 100mg/kg idasanutlin (MDM2i) as single agents or in combination. Body weight was measured at days 10, 17 and 24. Error bars represent SD of five mice (n=5).

F) Bar graph showing the body weight changes analyzed from figure S5E at day 24. Error bars represent SD of five mice (n=5).

Figure S6

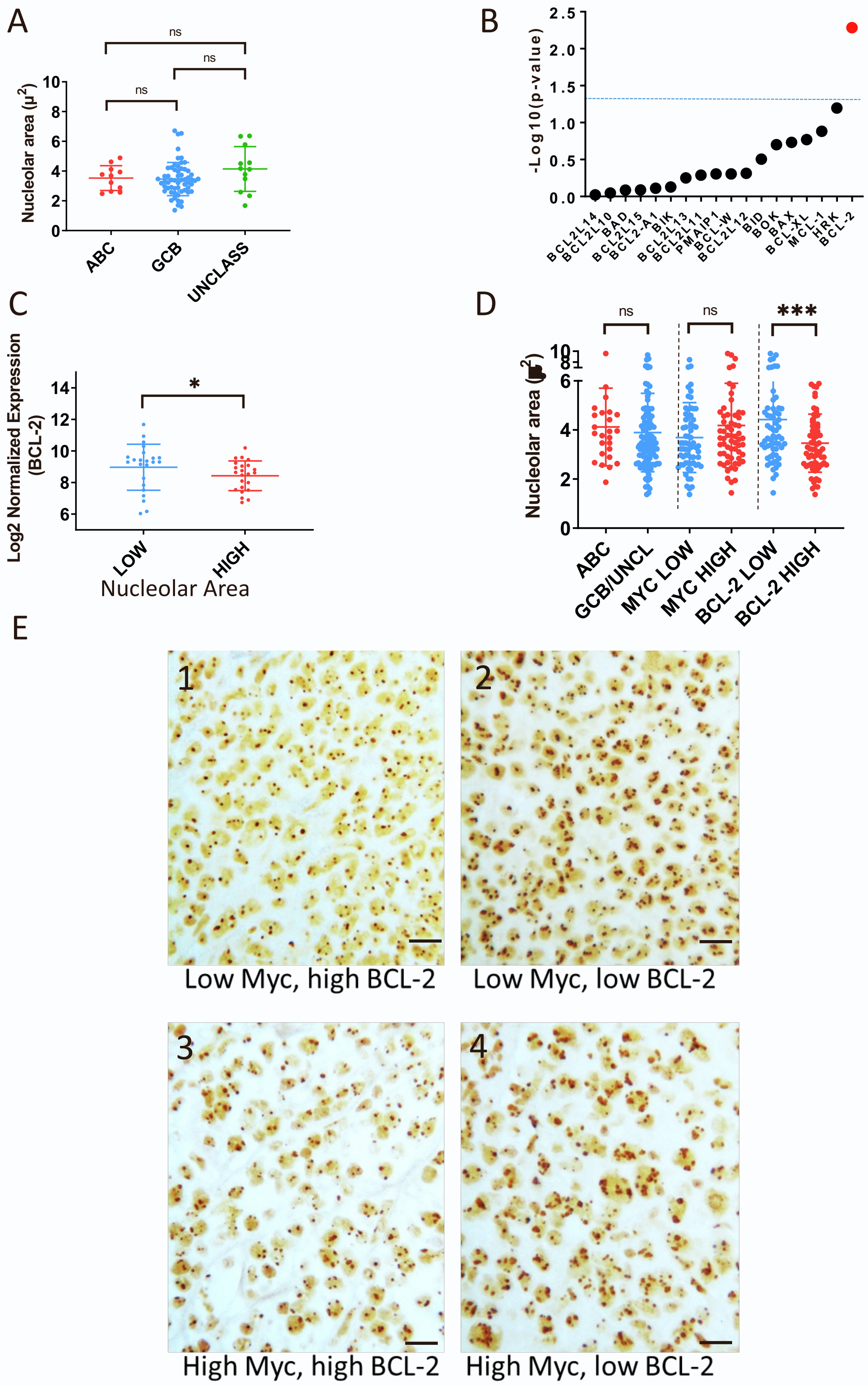
A
B
C
D
E
Nucleolar area (μ²)
ns
ABC
GCB
UNCLASS
-Log10(p-value)
BCL2L14
BCL2L10
BAD
BCL2L15
BCL2-A1
BIK
BCL2L13
BCL2L11
PMAIP1
BCL-W
BCL2L12
BID
BOK
BAX
BCL-XL
MCL-1
HRK
BCL-2
Log2 Normalized Expression (BCL-2)
*
LOW
HIGH
Nucleolar Area
***
GCB/UNCL
MYC LOW
MYC HIGH
BCL-2 LOW
BCL-2 HIGH
1
2
3
4
Low Myc, high BCL-2
Low Myc, low BCL-2
High Myc, high BCL-2
High Myc, low BCL-2

Figure S6: BCL-2 overexpression is associated with a decreased nucleolar area and adverse outcome in Diffuse Large B-cell Lymphoma

A) Dot plot graph showing values of nucleolar area as determined by quantitative image analysis of silver-stained nucleolar structures in patients' samples (exploratory cohort 1, N=83) according to the COO (as determined by T-GEP). Differences between groups were calculated with the Student's t-test.

B) Dot plot showing the the correlation between mRNA levels of several BCL-2 family members (as determined by T-GEP) and progression-free survival (PFS) in the exploratory cohort 1. As shown in the graph, among all BCL-2 family members, only *BCL-2* mRNA levels are significantly associated with PFS.

C) Dot plot graph showing levels of *BCL-2* mRNA expression in patients' samples (validation cohort 2, N=46) with low and high nucleolar area determined by quantitative image analysis of silver-stained nucleolar structures. The definition of "high" and "low" nucleolar area was based on the median value of the nucleolar area in the whole cohort. Student's t-test: *$p<0.05$.

D) Dot plot graph showing values of nucleolar area in patients' samples (exploratory + validation cohorts, N=129) according to the COO, *MYC* and *BCL-2* mRNA levels (as determined by T-GEP). Student's t-test: ***$p<0.005$.

E) Upper panels: nucleolar silver staining of DLBCL histological sections from two patients, both characterized by low *MYC* mRNA expression, but by a high (1) and a low (2) level of *BCL-2* mRNA, respectively. Lower panels: nucleolar silver staining of DLBCL histological sections from two patients, both characterized by a high *MYC* mRNA expression, but by a low (3) and a high (4) *BCL-2* mRNA expression, respectively. Note the very high amount of the silver-stained structures present in nuclei in A and C, some of which are very large in comparison with the relative low number of silver-stained nucleoli in B and D. Bar, 10 µm.

Figure S7

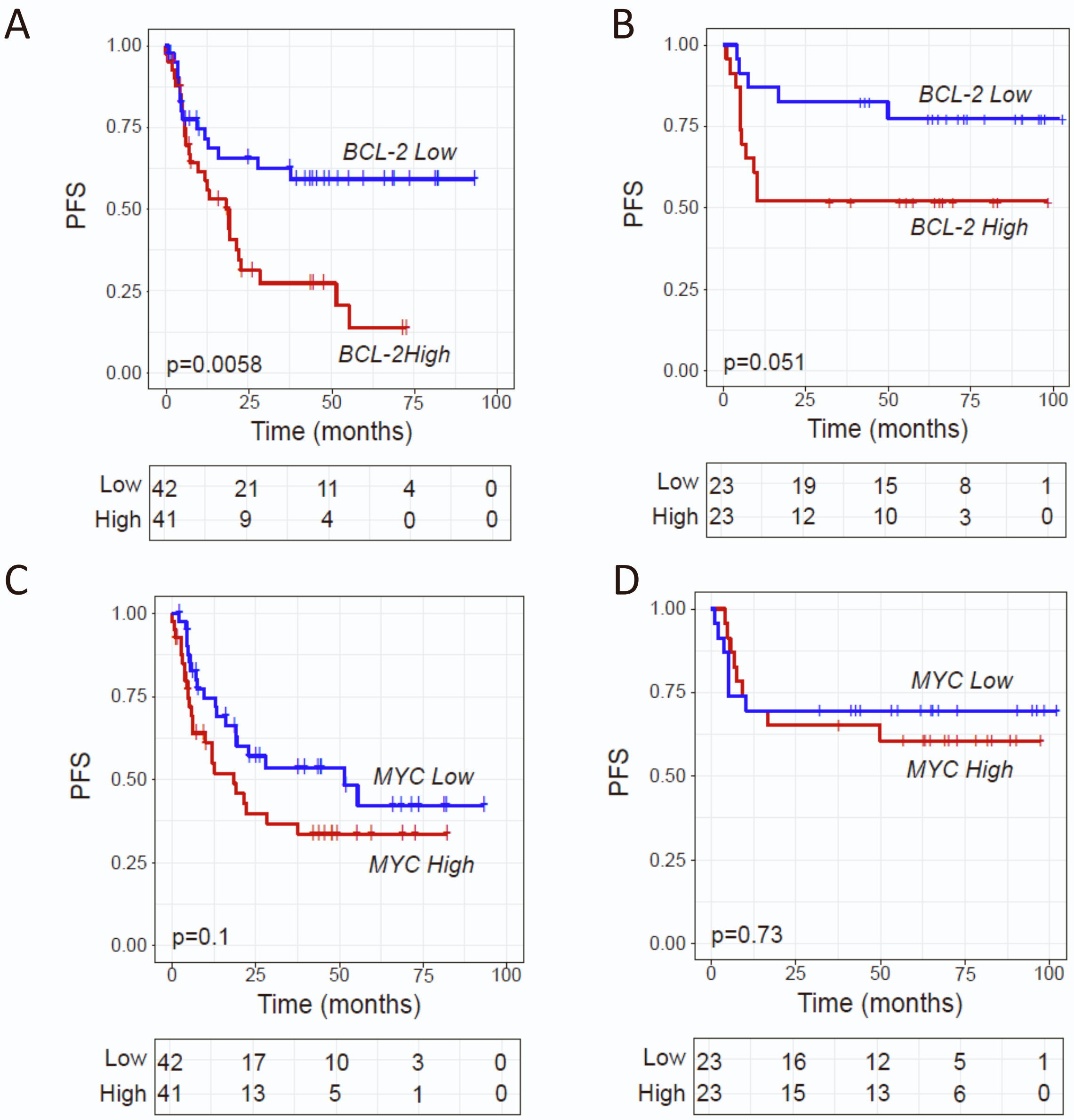
A
1.00
0.75
0.50
0.25
0.00
PFS
BCL-2 Low
BCL-2High
p=0.0058
0
25
50
75
100
Time (months)
Low 42 21 11 4 0
High 41 9 4 0 0
B
1.00
0.75
0.50
0.25
0.00
PFS
BCL-2 Low
BCL-2 High
p=0.051
0
25
50
75
100
Time (months)
Low 23 19 15 8 1
High 23 12 10 3 0
C
1.00
0.75
0.50
0.25
0.00
PFS
MYC Low
MYC High
p=0.1
0
25
50
75
100
Time (months)
Low 42 17 10 3 0
High 41 13 5 1 0
D
1.00
0.75
0.50
0.25
0.00
PFS
MYC Low
MYC High
p=0.73
0
25
50
75
100
Time (months)
Low 23 16 12 5 1
High 23 15 13 6 0

Figure S7: BCL-2 overexpression is associated with adverse outcome in DLBCL

A) Progression-free survival (PFS) curve of the exploratory cohort 1 (N=83) according to *BCL-2* mRNA levels. P-value was calculated with the log rank test.

B) Progression-free survival (PFS) curve of the validation cohort 2 (N=46) according to *BCL-2* mRNA levels. P-value was calculated with the log rank test.

C) Progression-free survival (PFS) curve of the exploratory cohort 1 (N=83) according to *MYC* mRNA levels. P-value was calculated with the log rank test.

D) Progression-free survival (PFS) curve of the validation cohort 2 (N=46) according to *MYC* mRNA levels. P-value was calculated with the log rank test.

Figure S8

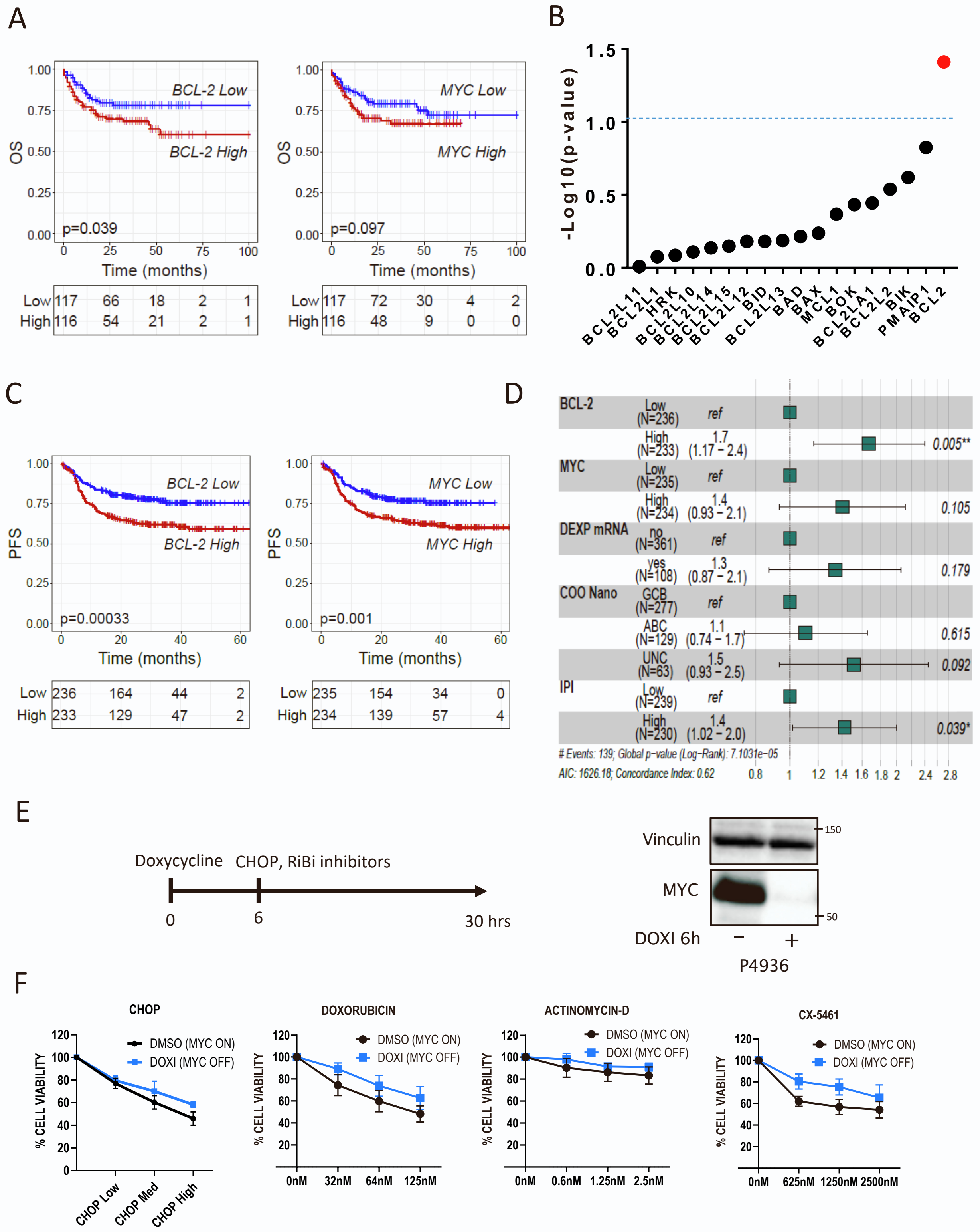
A
BCL-2 Low
BCL-2 High
OS
p=0.039
Time (months)
Low 117 66 18 2 1
High 116 54 21 2 1
MYC Low
MYC High
p=0.097
Low 117 72 30 4 2
High 116 48 9 0 0
B
-Log10(p-value)
BCL2L11 BCL2L1 HRK BCL2L10 BCL2L14 BCL2L15 BCL2L12 BID BCL2L13 BAD BAX MCL1 BOK BCL2A1 BCL2L2 BIK PMAIP1 BCL2
C
PFS
p=0.00033
Low 236 164 44 2
High 233 129 47 2
p=0.001
Low 235 154 34 0
High 234 139 57 4
D
BCL-2 Low (N=236) ref
High (N=233) 1.7 (1.17 - 2.4) 0.005**
MYC Low (N=235) ref
High (N=234) 1.4 (0.93 - 2.1) 0.105
DEXP mRNA no (N=361) ref
yes (N=108) 1.3 (0.87 - 2.1) 0.179
COO Nano GCB (N=277) ref
ABC (N=129) 1.1 (0.74 - 1.7) 0.615
UNC (N=63) 1.5 (0.93 - 2.5) 0.092
IPI Low (N=239) ref
High (N=230) 1.4 (1.02 - 2.0) 0.039*
Events: 139; Global p-value (Log-Rank): 7.1031e-05
AIC: 1626.18; Concordance Index: 0.62
E
Doxycycline
CHOP, RiBi inhibitors
0
6
30 hrs
Vinculin
MYC
DOXI 6h
P4936
F
CHOP
DOXORUBICIN
ACTINOMYCIN-D
CX-5461
% CELL VIABILITY
DMSO (MYC ON)
DOXI (MYC OFF)
CHOP Low
CHOP Med
CHOP High
0nM 32nM 64nM 125nM
0nM 0.6nM 1.25nM 2.5nM
0nM 625nM 1250nM 2500nM

Figure S8: BCL-2 overexpression is associated with adverse outcome in DLBCL (*in silico* validation cohorts)

A) Left: Overall survival (OS) curve of the Lenz cohort (N=233) according to *BCL-2* mRNA levels. P-value was calculated with the log rank test. Right: overall survival (OS) curve of the Lenz cohort (N=233) according to *MYC* mRNA levels. P-value was calculated with the Log rank test.

B) Dot plot showing the significance of the correlation between mRNA levels of several BCL-2 family members (as determined by T-GEP) and overall survival (OS) in the Lenz cohort. As shown in the graph, among all BCL-2 family members, only *BCL-2* mRNA levels are significantly associated with OS.

C) Left: progression-free survival (PFS) curve of the Sha cohort (N=469) according to *BCL-2* mRNA levels. P-value was calculated with the log rank test. Right: progression-free survival (PFS) curve of the Sha cohort (N=469) according to *MYC* mRNA levels. P-value was calculated with the log rank test.

D) Forest plot depicting multivariable analysis for PFS (Sha cohort).

E) Scheme of MYC overexpression experiments. Western blot displays levels of MYC protein expression after 6h incubation with 1µg/ml doxycycline in P-4936 cell line, where MYC expression is under the control of a tetracycline regulated repressible (Tet-OFF) promoter.

F) CTG assay showing the cytotoxic effects at 24h of 3 doses of CHOP (vincristine 1.5, 0.75, 0.37nM, doxorubicin 200, 100, 50nM, acrolein 6, 3, 1.5µM, methylprednisolone 50, 25, 12.5µM) or the indicated single agent RiBi inhibitors on cell viability in the presence or absence of MYC. Error bars represent SD of triplicate experiments (n=3).

Figure S9

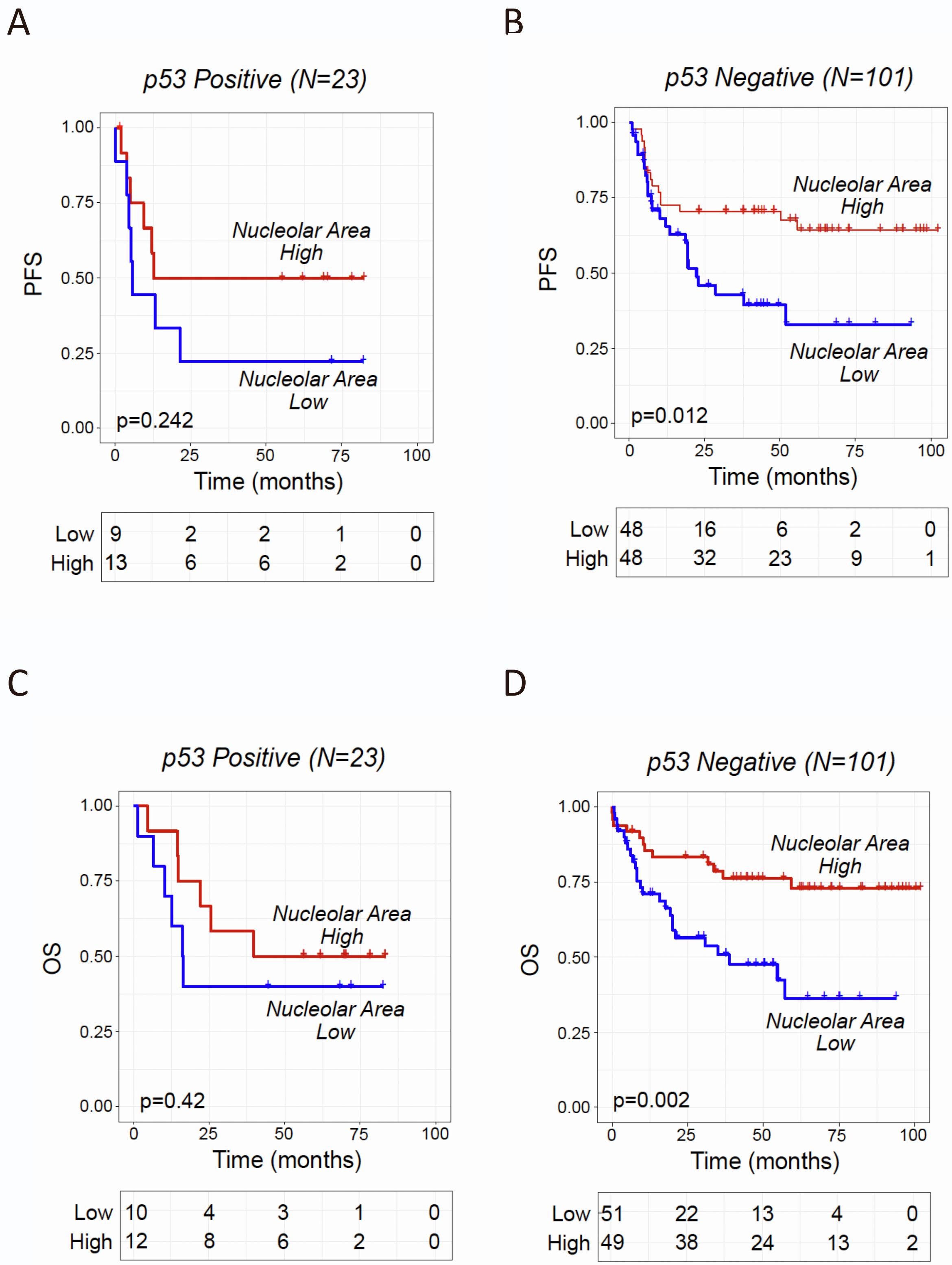
A
p53 Positive (N=23)
PFS
Nucleolar Area High
Nucleolar Area Low
p=0.242
Time (months)
Low 9 2 2 1 0
High 13 6 6 2 0
B
p53 Negative (N=101)
PFS
Nucleolar Area High
Nucleolar Area Low
p=0.012
Time (months)
Low 48 16 6 2 0
High 48 32 23 9 1
C
p53 Positive (N=23)
OS
Nucleolar Area High
Nucleolar Area Low
p=0.42
Time (months)
Low 10 4 3 1 0
High 12 8 6 2 0
D
p53 Negative (N=101)
OS
Nucleolar Area High
Nucleolar Area Low
p=0.002
Time (months)
Low 51 22 13 4 0
High 49 38 24 13 2

Figure S9: A reduced nucleolar area is an independent predictor of adverse outcome in DLBCL patients treated with standard anthracycline-based chemoimmunotherapy. Stratification according to the p53 status assessed by immunohistochemistry.

A) Progression-free survival (PFS) curve according to nucleolar size (nucleolar area high vs low) considering only patients with high p53 protein expression (≥50% positive cells, N=23) in the whole cohort (exploratory + validation). p-value was calculated with the Log rank test.

B) Progression-free survival (PFS) curve according to nucleolar size (nucleolar area high vs low) considering only patients with low p53 protein expression (≥50% positive cells, N=101) in the whole cohort (exploratory + validation). p-value was calculated with the Log rank test.

C) Overall survival (OS) curve according to nucleolar size (nucleolar area high vs low) considering only patients with high p53 protein expression (≥50% positive cells, N=23) in the whole cohort (exploratory + validation). p-value was calculated with the Log rank test.

D) Overall survival (OS) curve according to nucleolar size (nucleolar area high vs low) considering only patients with low p53 protein expression (≥50% positive cells, N=101) in the whole cohort (exploratory + validation). p-value was calculated with the Log rank test.

Supplementary Table S1.

Patient characteristics in AgNOR subgroups.

	AgNOR low	AgNOR high	P value
N° patients	65	64	-
Age<br>Median, range	64 (21-87)	56 (22-86)	0.09
COO T-GEP<br>GC<br>ABC<br>Unclassified	 50<br>9<br>6	 35<br>14<br>15	0.23 (ABC vs OTHER)
AgNOR area (μ^2)<br>Median, range	2.89 (1.37-3.55)	4.71 (3.6-9.58)	<0.001
aaIPI score<br>1-2<br>3	 45<br>20	 49<br>15	0.34
DEXP (IHC)<br>Yes<br>No	 15<br>50	 19<br>45	0.39
FISH status<br>MYC<br>BCL-2<br>BCL-6<br>DHIT	 5<br>7<br>18<br>4	 5<br>8<br>25<br>3	 0.97<br>0.75<br>0.43<br>0.71
DEXP T-GEP<br>Yes<br>No	 18<br>47	 17<br>47	0.88
BCL-2 mRNA<br>Median, range	9.73 (5.88-12.62)	9.032 (6.02-12-13)	0.001
MYC mRNA<br>Median, range	9.83 (7.52-11.92)	9.85 (7.32-13.97)	0.45
P53 (IHC)<br>Positive<br>Negative<br>NE	 11<br>52<br>2	 12<br>49<br>3	0.78
